# A programmed decline in ribosome levels governs human early neurodevelopment

**DOI:** 10.1038/s41556-025-01708-8

**Published:** 2025-08-04

**Authors:** Chunyang Ni, Yudong Wei, Barbara Vona, Dayea Park, Yulei Wei, Daniel A. Schmitz, Yi Ding, Masahiro Sakurai, Emily Ballard, Leijie Li, Yan Liu, Ashwani Kumar, Chao Xing, Shenlu Qin, Sangin Kim, Martina Foglizzo, Jianchao Zhao, Hyung-Goo Kim, Cumhur Ekmekci, Ehsan Ghayoor Karimiani, Shima Imannezhad, Fatemeh Eghbal, Reza Shervin Badv, Eva Maria Christina Schwaibold, Mohammadreza Dehghani, Mohammad Yahya Vahidi Mehrjardi, Zahra Metanat, Hosein Eslamiyeh, Ebtissal Khouj, Saleh Mohammed Nasser Alhajj, Aziza Chedrawi, Khushnooda Ramzan, Jamil A. Hashmi, Majed M. Alluqmani, Sulman Basit, Danai Veltra, Nikolaos M. Marinakis, Georgios Niotakis, Pelagia Vorgia, Christalena Sofocleous, Hane Lee, Won Chan Jeong, Muhammad Umair, Muhammad Bilal, César Augusto Pinheiro Ferreira Alves, Matthew Sieber, Michael Kruer, Henry Houlden, Fowzan S. Alkuraya, Elton Zeqiraj, Roger A. Greenberg, Can Cenik, Leqian Yu, Reza Maroofian, Jun Wu, Michael Buszczak

**Affiliations:** 1 Department of Molecular Biology, University of Texas Southwestern Medical Center, Dallas, TX USA; 2 Institute of Human Genetics, University Medical Center Göttingen, Göttingen, Germany; 3 Institute for Auditory Neuroscience and InnerEarLab, University Medical Center Göttingen, Göttingen, Germany; 4 Department of Molecular Biosciences, University of Texas at Austin, Austin, TX USA; 5 State Key Laboratory of Animal Biotech Breeding, College of Biological Sciences, China, Agricultural University, Beijing, China; 6 McDermott Center of Human Growth and Development, University of Texas Southwestern Medical Center, Dallas, TX USA; 7 Department of Physiology, University of Texas Southwestern Medical Center, Dallas, TX USA; 8 Department of Cancer Biology, Penn Center for Genome Integrity, Basser Center for BRCA, Perelman School of Medicine, University of Pennsylvania, Philadelphia, PA USA; 9 Astbury Centre for Structural Molecular Biology, School of Molecular and Cellular Biology, Faculty of Biological Sciences, University of Leeds, Leeds, UK; 10 State Key Laboratory of Organ Regeneration and Reconstruction, Institute of Zoology, Chinese Academy of Sciences, Beijing, China; 11 Department of Neurosurgery, Robert Wood Johnson Medical School, The State University of New Jersey, Rutgers, Piscataway, NJ USA; 12 DNA Laboratories, Istanbul, Turkey; 13 Centre for Neuromuscular Diseases, UCL Queen Square Institute of Neurology, London, UK; 14 Department of Medical Genetics, Next Generation Genetic Polyclinic, Mashhad, Iran; 15 Department of Pediatrics, Faculty of Medicine, Mashhad University of Medical Sciences, Mashhad, Iran; 16 Children’s Medical Center, Pediatrics Center of Excellence, Tehran University of Medical Sciences, Tehran, Iran; 17 Institute of Human Genetics, Heidelberg University, Heidelberg, Germany; 18 Abortion Research Centre, Yazd Reproductive Sciences Institute, Shahid Sadoughi University of Medical Sciences, Yazd, Iran; 19 Diabetes Research Center, Shahid Sadoughi University of Medical Sciences, Yazd, Iran; 20 Genetics of Non-Communicable Diseases Research Center, Zahedan University of Medical Sciences, Zahedan, Iran; 21 Department of Pediatrics, Shahid Sadoughi University of Medical Science, Yazd, Iran; 22 Department of Translational Genomics, Center for Genomic Medicine, King Faisal Specialist Hospital and Research Center, Riyadh, Saudi Arabia; 23 Medical Genetics Department, King Faisal Hospital Specialist & Research Centre, Riyadh, Saudi Arabia; 24 Department of Neurosciences, King Faisal Hospital Specialist & Research Centre, Riyadh, Saudi Arabia; 25 Department of Clinical Genomics, King Faisal Specialist Hospital and Research Centre, Riyadh, Saudi Arabia; 26 Department of Basic Medical Sciences, College of Medicine, Taibah University, Medina, Saudi Arabia; 27 Center for Genetics and Inherited Diseases, Taibah University, Medina, Saudi Arabia; 28 Department of Neurology, College of Medicine, Taibah University, Medina, Saudi Arabia; 29 Laboratory of Medical Genetics, Medical School, National and Kapodistrian University of Athens, St. Sophia’s Children’s Hospital, Athens, Greece; 30 Pediatric Neurology Department, Venizelion Hospital, Heraklion, Greece; 31 Agrifood and Life Sciences Institute, Hellenic Mediterranean University, Heraklion, Greece; 32 3billion, Seoul, South Korea; 33 Medical Genomics Research Department, King Abdullah International Medical Research Center (KAIMRC), King Saud bin Abdulaziz University for Health Sciences, Ministry of National Guard Health Affairs, Riyadh, Saudi Arabia; 34 Department of Life Sciences, School of Science, University of Management and Technology (UMT), Lahore, Pakistan; 35 Department of Biotechnology, Begum Nusrat Bhutto Women University, Sukkur, Pakistan; 36 Department of Radiology, Division of Neuroradiology, Children’s Hospital of Philadelphia, Philadelphia, PA USA; 37 Department of Radiology, Neuroradiology Division, Boston Children’s Hospital, Harvard Medical School, Boston, MA USA; 38 Pediatric Movement Disorders Program, Barrow Neurological Institute, Phoenix Children’s Hospital, Phoenix, AZ USA; 39 Department of Neuromuscular Diseases, UCL Institute of Neurology, Queen Square, London, UK; 40 Beijing Institute for Stem Cell and Regenerative Medicine, Beijing, China; 41 Hamon Center for Regenerative Science and Medicine, University of Texas Southwestern Medical Center, Dallas, TX USA; 42 Cecil H. and Ida Green Center for Reproductive Biology Sciences, University of Texas Southwestern Medical Center, Dallas, TX USA

**Keywords:** Stem-cell differentiation, Disease model, Mechanisms of disease

## Abstract

Many neurodevelopmental defects are linked to genes involved in housekeeping functions, such as those encoding ribosome biogenesis factors. How reductions in ribosome biogenesis can result in tissue- and developmental-specific defects remains unclear. Here we describe variants in the ribosome biogenesis factor *AIRIM/C1orf109* that are primarily associated with neurodevelopmental disorders. Using human cerebral organoids in combination with proteomic, single-cell RNA sequencing and single-organoid translation analyses, we identify a previously unappreciated drop in protein production during early brain development. We find that ribosome levels decrease during neuroepithelial differentiation, making differentiating cells particularly vulnerable to perturbations in ribosome biogenesis during this time. Reduced ribosome availability more profoundly impacts the translation of specific transcripts, disrupting both survival and cell fate commitment of transitioning neuroepithelia. Enhancing mTOR activity suppresses the growth and developmental defects associated with *AIRIM/C1orf109* variants. This work provides evidence for the functional importance of regulated changes in global protein synthesis capacity during cellular differentiation.

## Abstract (web summary)

Ni, Wei, Vona and colleagues use human brain organoids to dissect patient AIRIM variants associated with neurodevelopmental features. A subset of variants impaired ribosome production and protein synthesis, and delayed radial glial cell specification.

## Main

Human brain development depends on the coordinated action of various signalling pathways, coupled with cell-specific and stage-specific gene expression, to specify a diverse range of cell types^1^. During the initial stages of brain development, neuroepithelial (NE) cells undergo proliferation and differentiate into radial glial (RG) progenitors, intermediate progenitors and outer RG progenitors. Progenitor cells in the dorsal region differentiate into excitatory neurons, whereas those in the ventral region give rise to interneurons that subsequently migrate to the dorsal cortex. It is well known that initial cell fate decisions are influenced by the differential expression of crucial transcription factors^2,3^. However, the role of post-transcriptional regulation in early brain development remains less explored.

The study of neurodevelopmental disorders (NDDs) has provided key insights into the mechanisms that govern these early steps in human brain development. NDDs are a broad spectrum of disorders that affect more than 4.7% of children globally and include intellectual disabilities, seizures, defects in sensory perception and in extreme cases, microcephaly^4^. NDDs are most frequently caused by genetic lesions. Notably, only a small fraction of these mutations affect genes that have a primary role in human brain development^5^. Rather, many NDD-associated allelic variants map to genes involved in general cellular housekeeping functions. How mutations in these essential housekeeping genes lead to tissue-specific and developmentally distinct phenotypes remains poorly understood.

Mutations in ribosome-related genes cause a group of related diseases called ribosomopathies, which include Diamond Blackfan anaemia (DBA), Treacher Collins syndrome (TCS), X-linked dyskeratosis congenita (DC) and cartilage hair hypoplasia (CHH)^6,7^. The phenotypes associated with ribosomopathies vary widely. For example, patients with DBA, which is linked with mutations in several genes, including *RPS19*, *RPL5* and *TSR2*, primarily present with severe anaemia and only occasionally suffer from intellectual disabilities^8^. By contrast, TCS, which is associated with loss of the Pol I factor Treacle Ribosome Biogenesis Factor 1 (TCOF1), results in craniofacial malformations^9^. Recent results indicate that mutations in Ribosomal RNA Processing 7 Homolog A, RRP7A and dysfunction of ribosome quality control mechanisms are also associated with neurological disorders^10,11^. Disruption of ribosome biogenesis or its function often results in a nucleolar stress response that induces p53 activity. Genetic ablation of p53 can suppress many of the phenotypes associated with models of ribosomopathies^12–14^. However, the molecular and cellular mechanisms responsible for the tissue specificity of these diseases remain incompletely understood.

We recently characterized a complex composed of AFG2 Interacting Ribosome Maturation Factor (AIRIM), also known as C1orf109, AFG2 AAA ATPase Homolog A (AFG2A), also known as SPATA5, AFG2 AAA ATPase Homolog B (AFG2B), also known as SPATA5L1 and Cyclin-Dependent Kinase 2 Interacting Protein (CINP), which promotes the recycling of Ribosomal L24 Domain Containing 1 (RSL24D1) from cytoplasmic pre-60S ribosomal subunits back to the nucleolus^15^. This complex is also referred to as the 55LCC (SPATA5–SPATA5L1–C1orf109–CINP) complex^16^. SPATA5/AFG2A is the human orthologue of yeast Drg1, which plays a well characterized role in ribosome biogenesis^17^. Additional studies have further implicated human AFG2A and AFG2B in ribosome production^18,19^. The AFG2 complex also plays a role in replisome proteostasis^16^ and in regulating mitochondrial function^20^. Of note, human genetic studies have identified a growing number of allelic variants in *AFG2A*, *AFG2B* and *CINP* that are specifically associated with a range of NDDs, including intellectual disabilities, seizures, hearing loss and microcephaly^20–26^. Here, we identify variants in *AIRIM* associated with a similar range of neurological phenotypes. Thus, unlike variants linked with DBA and other ribosomopathies, disruptions in the 55LCC complex primarily result in NDDs. These observations beg the question of whether phenotypes associated with these variants represent a ribosomopathy, and if so, how do disruptions in 60S biogenesis result in brain-specific abnormalities.

NDDs are not always recapitulated in mouse models. To overcome this experimental barrier, many groups have turned to using human induced pluripotent stem (iPS) cell-derived brain organoids^27–29^. These organoid models have proven useful for studying human brain development. Transcriptional and epigenetic analyses indicate that brain organoids in 3D culture recapitulate many of the same developmental processes and gene expression programmes that occur during fetal development in vivo^2,30^. Moreover, these models have been extremely useful for characterizing mechanisms involved in various neurodevelopmental diseases, including microcephaly and autism^29,31^.

In this study, we have characterized *AIRIM* and *AFG2B* variants using cerebral organoids, which provides evidence that the dynamic regulation of ribosome levels is critical for early neurodevelopment. Defects associated with patient variants in both *AIRIM* and *AFG2B* can be traced back specifically to NE differentiation. These variants cause reductions in the translation of a specific subset of messenger RNAs encoding components of protein synthesis machinery, factors needed for early cell fate specification, and regulators of mitochondrial function. This decrease in the protein expression of specific factors causes a delay in the NE to RG cell transition. We found that genetically or pharmacologically increasing mTOR signalling alleviates the growth, enhanced cell death and cell-fate specification phenotypes observed in organoids that carry a pathogenic *AIRIM* variant. These findings highlight the importance of the stage-specific regulation of protein synthesis and ribosome availability in the developing human nervous system.

## Results

### *AIRIM* variants linked with neurodevelopmental disorders

**Fig. 1:**
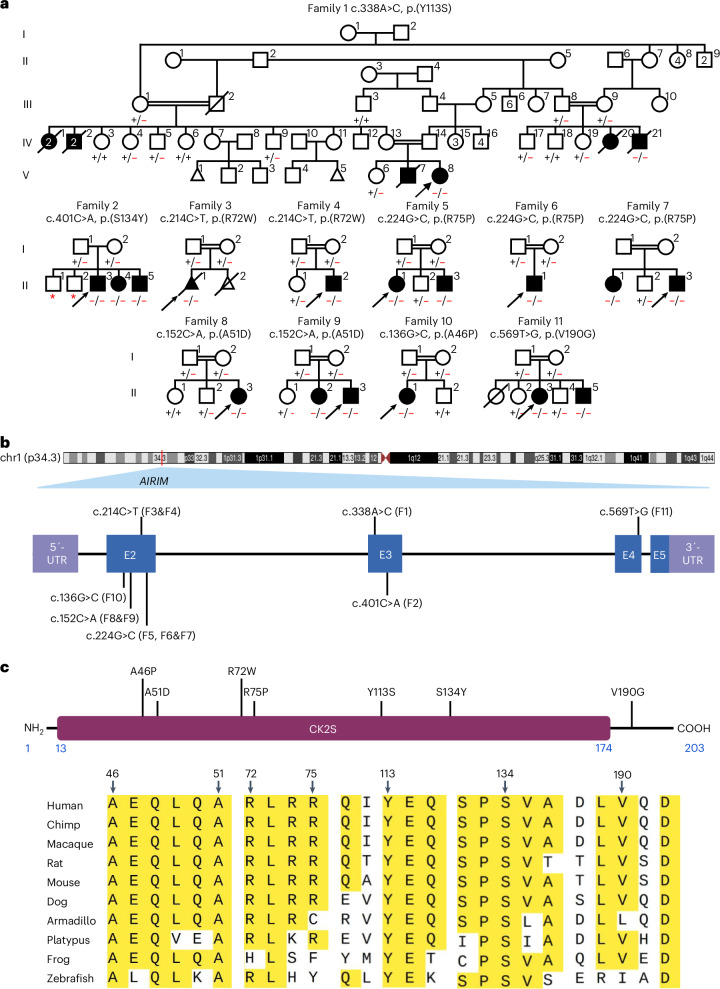
Family pedigrees, variant schematic on gene and protein level and amino acid conservation of *AIRIM* substitutions. **a**, Pedigrees showing segregation of the variants. Affected and unaffected individuals are indicated by filled and open and filled symbols, respectively. Probands are marked with arrows. Double lines indicate consanguinity. Red asterisks represent a nonhomozygous genotype. Segregation results are shown by the presence of a red minus symbol for the variant or a black plus symbol representing the reference allele, where −/− and +/+ represent a homozygous variant or wild-type genotype, respectively, and +/− represents a heterozygous genotype. **b**, Schematic representation of the gene and protein positions of *AIRIM* variants. *AIRIM* is located on chromosome 1 at cytogenetic position p34.3 (top). Below: the genetic variants are mapped to the NM_017850.3 transcript of *AIRIM*. **c**, *AIRIM* variants shown on the protein level. The casein kinase II substrate (CK2S) region is shown in maroon with the corresponding amino acid coordinates of the protein and domain in blue (top). Variants are labelled by family codes. Alignment of multiple *AIRIM* orthologues with amino acid substitutions marked with arrows (bottom).

**Fig. 2:**
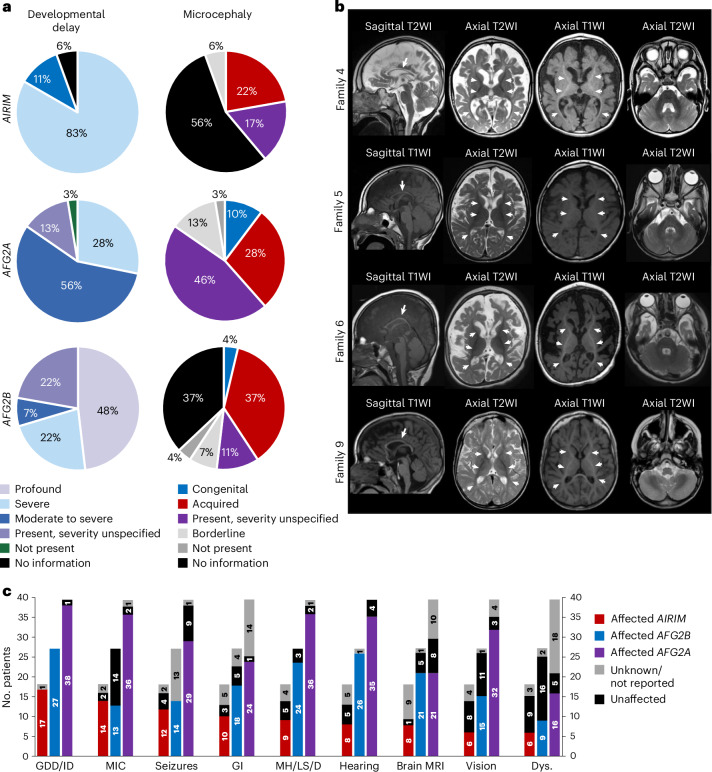
Clinical features of the affected individuals with biallelic *AIRIM* variants. **a**, Summary of global developmental delay and microcephaly in aggregated published individuals to date. **b**, Brain MRI of the 1-year and 11-month-old proband in family 4 (II:2). Sagittal T2-weighted image (WI), axial T2WI and axial T1WI showing atrophy in the supratentorial compartment of the brain, including a diffusely thin corpus callosum (arrow) and enlargement of the extra-axial spaces. Abnormal T2WI hyperintensity of the deep and superficial white matter with relatively normal myelination appearance along the anterior and posterior limbs of the internal capsules, as well as the optic tracts (arrowheads). Brain MRI of the sibling of the proband in family 5 (II:3) showing severe atrophy in the supratentorial compartment of the brain, including a diffusely thin corpus callosum (arrow) and enlargement of the extra-axial spaces. Abnormal T2WI hyperintensity of the deep and superficial white matter with relatively normal myelination appearance along the anterior and posterior limbs of the internal capsules, as well as the optic tracts (arrowheads). It is also important to note the relatively normal appearance of the overall structures of the posterior fossa with normal volume, morphology and myelination appearance of the cerebellum. Brain MRI of the proband in family 6 (II:1) at 6 months old; sagittal T1WI, axial T2WI and axial T1WI. Severe atrophy in the supratentorial compartment of the brain, including a diffusely thin corpus callosum (arrows) and diffuse enlargement of the extra-axial spaces with bilateral under-opercularization of Sylvian fissures. Abnormal T2WI hyperintensity of the deep and superficial white matter with relatively normal myelination appearance along the anterior and posterior limbs of the internal capsules, as well as the optic tracts (arrowheads). It is also important to note the relatively normal appearance of the overall structures of the posterior fossa with normal volume, morphology and myelination appearance of the cerebellum. Brain MRI of the proband in family 9 (II:3) showing severe atrophy in the supratentorial compartment of the brain, including a diffusely thin corpus callosum (arrow) and enlargement of the extra-axial spaces. Abnormal T2WI hyperintensity of the deep and superficial white matter with relatively normal myelination appearance along the anterior and posterior limbs of the internal capsules, as well as the optic tracts (arrowheads). It is also important to note the relatively normal appearance of the overall structures of the posterior fossa with normal volume, morphology and myelination appearance of the cerebellum. **c**, Bar graphs summarizing the comparative proportions of various clinical findings in individuals with *AIRIM*, *AFG2B* and *AFG2A* variants. Dys., dysmorphic facial features; GDD/ID, global developmental delay/intellectual disability; GI, gastrointestinal issues; MH/LS/D, muscular hypotonia/limb spasticity/dystonia; MIC, microcephaly.

Mutations in genes involved in ribosome biogenesis can disrupt the development and function of specific tissues, while leaving others unaffected, a phenomenon that remains incompletely understood. We hypothesized that identifying variants in ribosome biogenesis factors specifically associated with NDDs would shed light on how disturbances in translation can selectively affect early brain development. Through an extensive international collaboration, we identified a cohort of 11 unrelated families with overlapping clinical features, encompassing 18 individuals, 17 of whom were born. Affected individuals presented with moderate-to-severe or severe global developmental delay/intellectual disability (17 of 17) and never achieved developmental milestones. The majority of individuals from whom information was available concomitantly showed muscular hypotonia accompanied by limb spasticity and dystonia (each 11 of 17), microcephaly (14 of 17), as well as hearing (9 of 17) and vision impairment (6 of 17) and dysmorphism (6 of 17). Infantile seizures were reported in 12 individuals and further characterized in 11 as generalized tonic and clonic (4 of 11), myoclonic (3 of 11), Lennox–Gastaut syndrome (2 of 11), tonic–clonic (1 of 11) and infantile spasm (1 of 11). Pedigrees, clinical details and variant characteristics identified in these families are provided (Figs. 1 and 2 and Supplementary Tables 1 and 2). The neuroimaging analysis revealed severe supratentorial brain atrophy with diffusely thin corpus callosum and ex-vacuum dilatation of the lateral ventricles along with under-opercularization of the Sylvian fissures, while the brainstem and cerebellum were relatively preserved (Fig. 2b and Supplementary Tables 1 and 2). Additionally, abnormal signal intensity was observed in both the deep and superficial white matter, indicating impaired myelination with hypomyelination appearance. However, normal myelination was observed in the limbs of the internal capsules, optic tracts and posterior fossa structures.

Using exome sequencing and homozygosity mapping, we identified a homozygous missense variant c.338A>C; p.(Tyr113Ser) in *AIRIM* residing in a ~12-Mb run of homozygosity (ROH) (chr1:26,392,624–38,748,480) from studying an extended consanguineous family (family 1) with multiple affected individuals. Subsequent sequencing of either proband or parent–child trios, along with extensive data sharing and screening large sequencing disease databases, identified five other homozygous missense variants: c.401C>A; p.(Ser134Tyr); c.214C>T; p.(Arg72Trp); c.224G>C; p.(Arg75Pro); c.152C>A; p.(Ala51Asp); c.136G>C; p.(Ala46Pro); and c.569T>G; p.(Val190Gly) (Fig. 1a and Supplementary Table 1). ROH intervals were analysed in unrelated families with the same variants. Families 3 and 4, who each have a c.214C>T; p.(Arg72Trp) variant, presented ROH intervals spanning different sizes (family 3: chr1:36,859,876–61,875,485 with a size of ~25 Mb; family 4: chr1:27,362,142–65,379,359 with a size of ~38 Mb) and shared an overlapping ROH spanning ~25 Mb (chr1:36,859,876–61,875,485) that included the *AIRIM* variant. Families 5–7, who were identified with the c.224G>C; p.(Arg75Pro) variant, have the same ROH interval spanning ~874 kb (chr1:37,977,771–38,851,959). Similarly, families 8 and 9, with the c.152C>A; p.(Ala51Asp) variant shared a common ROH (chr1:35,925,860–38,226,963) with a size of ~2.3 Mb. The shared ROH coordinates of the c.224G>C; p.(Arg75Pro) and c.152C>A; p.(Ala51Asp) variants and same self-reported ethnicities of these families (Saudi Arabian and Greek; Supplementary Table 2) suggest these as founder alleles. All variants segregated within the families, and they are either entirely absent or present at an extremely low allele frequency in sequence variant databases. In addition, c.65A>G; p.(Glu22Gly); c.97C>T; p.(Arg33Trp); c.187C>A; p.(Leu63Ile) were identified as predicted nonpathogenic missense variants based on the presence of five or more individuals homozygous for these variants in gnomAD.

**Extended Data Fig. 1:**
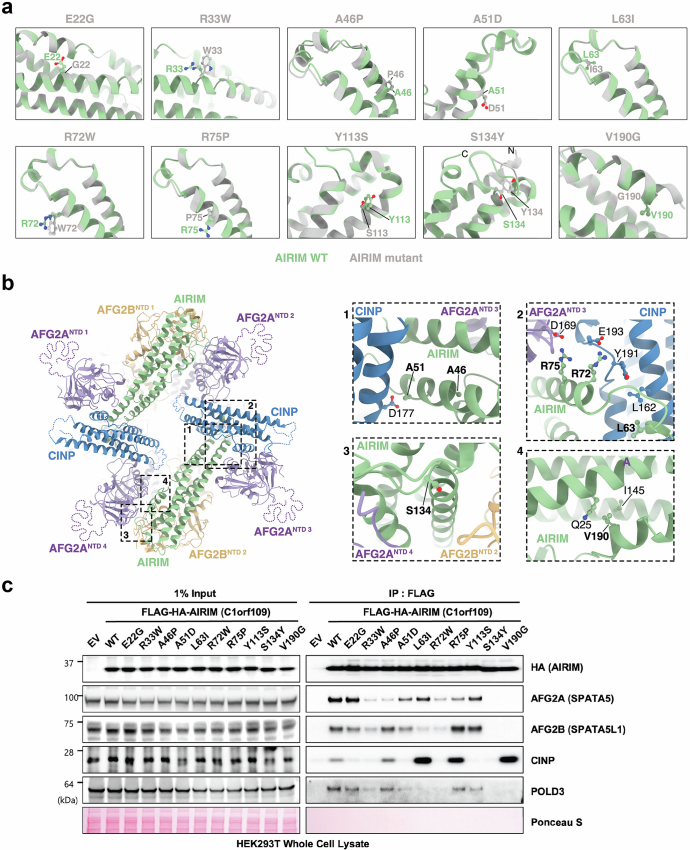
Structural and functional analysis of AIRIM variants. **a** Close-up views and structural details of AIRIM residues mutated in neurodevelopmental syndromes. Wild-type and mutant AIRIM variants predicted by AlphaFold 3 are shown in cartoon representation, and coloured light green or grey respectively. Mutated residues are shown as ball & sticks; N- and C-termini are indicated. AFG2A, AFG2B and CINP subunits have been removed for clarity. **b**. Top view of the 55LCC structure (PDBid: 8RHN), showing the arrangement of the 55LCC “Lid” unit. Cartoon models of the AIRIM–CINP heterodimer, and of the N-termini of AFG2A and AFG2B are coloured in light green, blue, purple and light orange respectively. Missing loops within each CINP protomer or unstructured regions in the AFG2A N-termini are indicated with dashed lines; superscript numbers refer to the AFG2A and AFG2B subunits. Dashed black rectangles highlight regions where AIRIM mutants and interacting residues are located (left). Close-up views and structural details of AIRIM residues mutated in neurodevelopmental syndromes. Disease-related and interacting residues are labelled in bold and roman respectively and shown as ball & sticks (right) NTD = N-terminal domain. **c**. Western blot of FLAG-immunoprecipitation (IP) and input samples from HEK293T transiently expressing FLAG–HA-AIRIM variants, empty vector (EV), or wild-type (WT). n = 3 biological replicates. Source data

Recent work has provided key insights into the structure of the 55LCC complex^16^; 55LCC is composed of two AIRIM and CINP heterodimers sitting on top of a heterohexameric ATPase ring formed by four copies of AFG2A and two copies of AFG2B. AlphaFold 3 predictions of disease-related AIRIM variants showed that seven of the AIRIM mutations result in strong (A46P, L63I, R75P and S134Y) or moderate (A51D, R72W and V190G) structural distortion, while the remaining three (E22G, R33W and Y113S) do not cause any structural changes (Extended Data Fig. 1a). We mapped the positions of these mutants in the 55LCC structure^16^ revealing that AIRIM A46, A51, L63, R72 and R75 localize in the proximity of, or at the AIRIM–CINP interface (Extended Data Fig. 1b). By contrast, AIRIM S134 is located in loop 4, near the AIRIM–AFG2B interface, whereas V190 localizes within α-helix 5 (α5) (Extended Data Fig. 1b). Although S134 of AIRIM does not interact with neighbouring amino acids, V190 contacts residues Q25 and I145 in helices α1 and α3, respectively (Extended Data Fig. 1b). These observations suggest that AIRIM disease-associated mutations will likely impact its stability or affect its interactions with CINP and AFG2A. To test these hypotheses, we transiently expressed Flag–HA-tagged AIRIM constructs carrying each of the allelic variants in HEK293T cells, immunoprecipitated the corresponding AIRIM proteins and probed the pellets for the presence of AFG2A, AFG2B, CINP and its substrate POLD3 (Extended Data Fig. 1c). Several AIRIM variants were consistent with structural predictions. For example, the AIRIM A51D and R72W mutations showed defects in interaction with CINP, whereas the R75P mutation impaired interaction with AFG2A (Extended Data Fig. 1c). This analysis revealed that many of the AIRIM variants disrupt the ability of the protein to interact with other components of the 55LCC complex, providing insights into how each of these alleles disrupt complex formation and function.

### Organoids as model of brain ribosomopathies

**Fig. 3:**
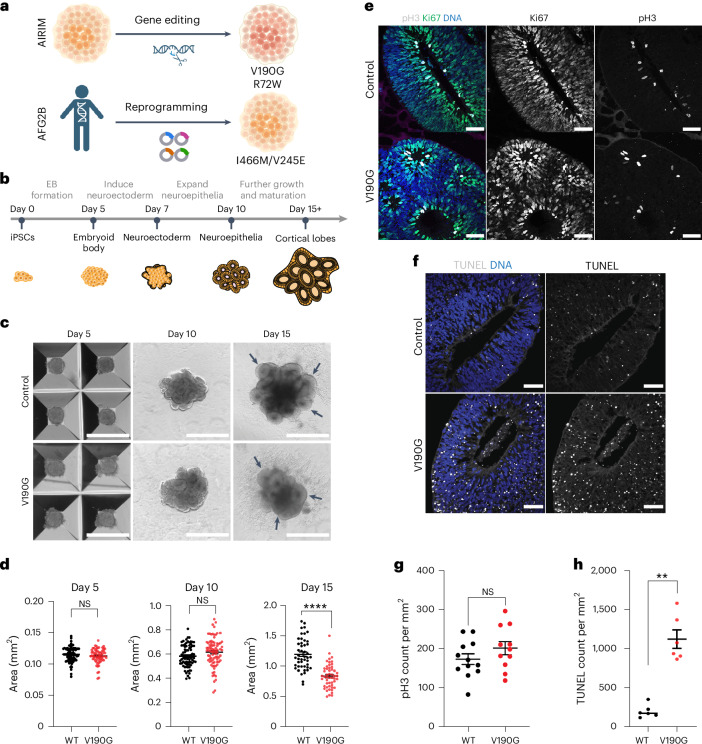
The NDD-associated *AIRIM*^*V190G*^ variant causes growth defects at a specific stage of cerebral organoid development. **a**, Schematic explaining the origin of the variant samples used in this study. **b**, Schematic of the timeline for generating brain organoids from pluripotent stem cells. **c**, Bright-field images of control and mutant (V190G) organoids at days 5, 10 and 15. Black arrows indicate NE buds, which seem less organized and elongated in the mutant at day 15. Scale bar, 1 mm. **d**, Quantification of bright-field images at days 5 (left), 10 (middle) and 15 (right) show that control neural tissue is enlarged relative to mutant (V190G); ****P < 0.0001, unpaired two-sided *t*-test with Welch’s correction, day 5, *n* = 73 control EBs 62 mutant EBs from three independent batches; day 10, *n* = 77 control organoids 84 mutant organoids from four independent batches; day 15, *n* = 46 control organoids 51 mutant organoids from three independent batches. Error bars show s.e.m. *P* = 0.8776 (day 5), *P* = 0.1128 (day 10), *P* = 2.515 × 10^−11^ (day 15). NS, not significant. **e**, Representative images showing the proliferation marker pH3 (grey) and Ki67 (green) signal in control (WT) and mutant (V190G) day 15 organoids. Scale bar, 50 μm. **f**, Representative images showing TUNEL labelling in control and mutant (V190G) day 15 organoids. Mutant organoids exhibit a higher TUNEL signal. Scale bar, 50 μm. **g**, Quantification of pH3 signal of individual NE buds at day 15 control (WT) and mutant (V190G) organoids. Unpaired two-sided *t*-test with Welch’s correction. *n* = 12 control and 11 mutant imaged regions from six organoids from two independent batches. Error bars show s.e.m. **h**, Quantification of TUNEL signal of individual NE bud showing that mutant organoids exhibit a higher TUNEL signal. TUNEL counts were normalized to the area of the imaged bud. **P < 0.01, unpaired two-sided *t*-test with Welch’s correction, *P* = 0.0022, *n* = 6 control *n* = 6 mutant imaged regions from two independent batches. Error bars show s.e.m. The image in **a** was made using BioRender. Source data

**Extended Data Fig. 2:**
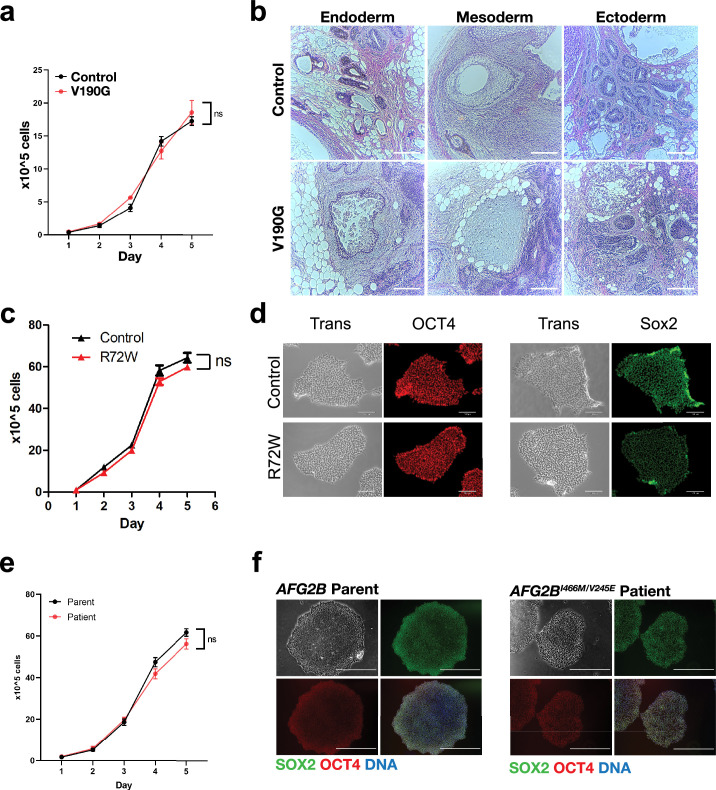
Proliferation and pluripotency assays of iPS cell lines. **a** Growth of parental and *AIRIM*^*V190G*^ lines. Error bars are SEM. **b**. Teratoma assay of SCVI274 (control) and the derived *AIRIM*^*V190G*^ mutant (V190G) lines. Scale bar, 200 μm. **c**. Growth of parental and *AIRIM*^*R72W*^ lines. Error bars are SEM. **d**. Immunofluorescence image of pluripotency markers SOX2 (green) and OCT4 (red) of *AIRIM*^*R72W*^ iPS cell clones. Scale bar, 1 mm. **e**. Growth of parental and *AFG2B* patient lines. Error bars are SEM. **f**. Immunofluorescence image of pluripotency markers SOX2 (green) and OCT4 (red) of iPS cell clones derived from the AFG2B patient and parental control fibroblasts. Scale bar, 1 mm. Two-way RM ANOVA. n = 4 individual wells quantified per genotype, per time point for panels **a**,**c**,**e**. Source data

**Extended Data Fig. 3:**
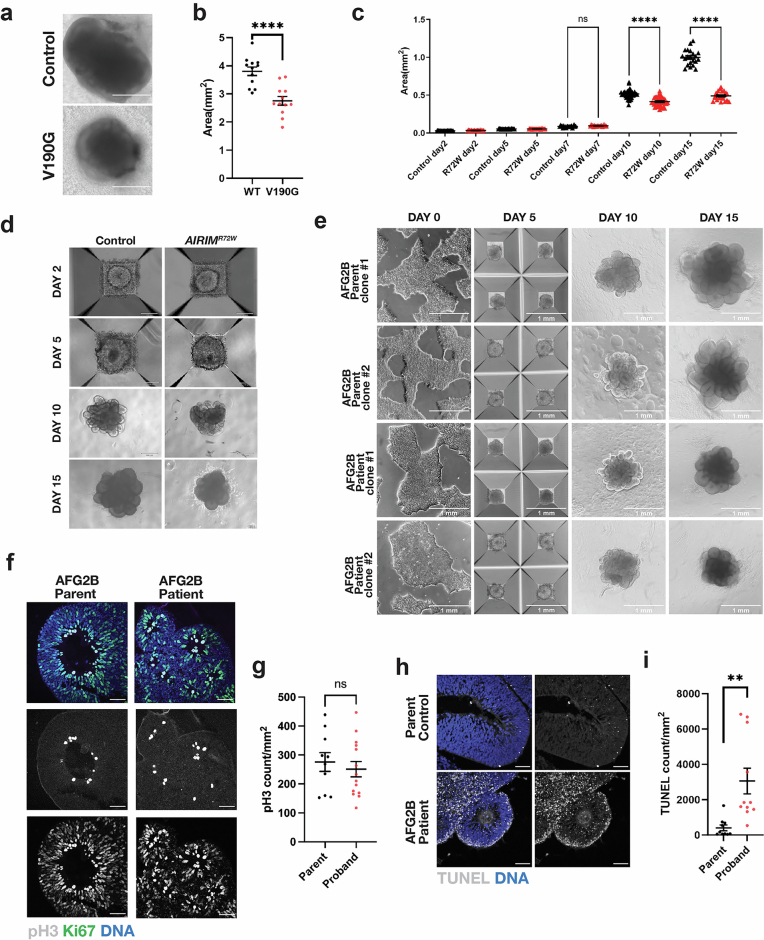
Disease associated *AIRIM* and *AFG2B* variants induces stage-specific growth defects of cerebral organoids. **a** Bright-field images of SCVI274 (control) and the *AIRIM*^*V190G*^ mutant (V190G) organoids at day 30. Note both control and mutant organoids show cortical plate structure. Scale bar, 1 mm. **b**. Quantification of organoid size in bright-field images at day 30. Control neural tissue is enlarged relative to mutant (V190G); ****P < 0.0001, Unpaired two-sided t-test with Welch’s correction, n = 12 control organoids and 12 mutant organoids from 2 independent batches, *P* = 7.371387e-05, error bars are SEM. **c**. Quantification of organoid size in bright-field images at days 2, 5, 7, 10 and15 (R72W). ****P(Day 10) = 2.28472e-11, ****P(Day 15) = 1.99452e-23. Dunettes multiple comparison Unpaired t-test with Welch’s correction (two-sided). n (Day 2) = 23 control EBs 24 mutant organoids from 3 independent batches, n (Day 5) = 23 control organoids 23 mutant organoids from 3 independent batches, n (Day7) = 21 control organoids 22 mutant organoids from 3 independent batches, n (Day 10) = 39 control organoids 37 mutant organoids from 3 independent batches, n (Day 15) = 21 control organoids 23 mutant organoids from 3 independent batches, error bars are SEM. **d**. Control and *AIRIM*^*R72W*^ derived EBs and organoids at days 2, 5, 10, and 15. Scale bar in day 2 and day 5, 130 μm, Scale bar in day 10 and day 15, 320 μm. 3 independent batches were performed. **e**. Parental control and *AFG2B* patient derived EBs and organoids at days 5, 10, and 15. Scale bar, 1 mm. Note organoids from patient derived iPS cells exhibit reduced growth and less elongated neuroepithelial buds on day 15. 2 independent batch were performed. **f**. Representative images showing the proliferation marker phospho-Histone 3 (pH3, grey) and Ki67 (green) signal in control (parent) and mutant (AFG2B patient) day 15 organoids. Scale bar, 50 μm. **g**. Quantification of pH3 signal of individual neuroepithelial bud at day 15 control (parent) and mutant (*AFG2B* patient) organoids. Unpaired two-sided t-test with Welch’s correction. n = 10 control and 14 mutant imaged regions from 2 independent batches. *P* = 0.8875, error bars are SEM. **h**. Representative images showing the TUNEL signal in control (parent) and mutant (*AFG2B* patient) day 15 organoids. Note mutant organoids exhibit significantly more TUNEL signal. Scale bar, 50 μm. **i**. Quantification of TUNEL signal of individual neuroepithelial bud showing mutant organoids exhibit more TUNEL signal. TUNEL counts were normalized to the area of the imaged bud. **P < 0. 01, Unpaired two-sided t-test with Welch’s correction. n = 10 control and 11 mutant imaged regions from 2 independent batches. *P* = 0.0044, error bars are SEM. Source data

**Extended Data Fig. 4:**
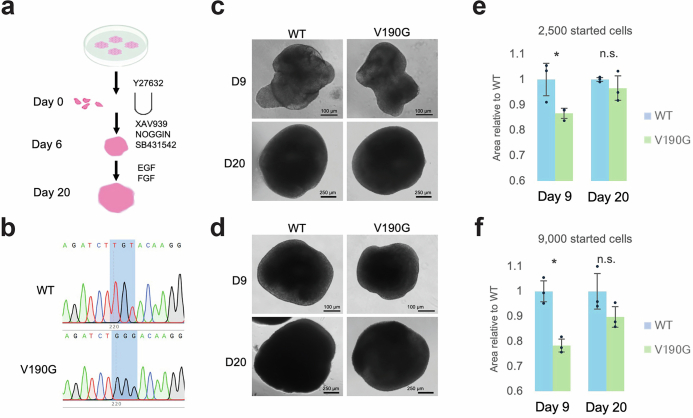
Additional *AIRIM*^*V190G*^ variant clone derived from H9 cells exhibits similar growth defect as other 55LCC complex members. **a** Schematic illustrating the main stages of the method for generating human dorsal forebrain organoids^75^ from H9 hESCs. **b**. Sanger sequencing of *C1orf109* V190G mutation in mutation cell line. **c**. Bright-field images of brain organoid at day 9 and day 20, with 2500 WT and V190G start cells respectively. **d**. Bright-field images of brain organoid at day 9 and day 20, with 9000 WT and V190G start cells, respectively. **e**. Measuring area of brain organoid generated from 2,500 WT and V190G cells (day 9 *P* = 0.047, day 20 *P* = 0.385, n = 3 biological replicates, unpaired, 2-tailed Student’s t-test), respectively. Data are presented as mean ± SD. **f**. Measuring area of brain organoid generated from 9,000 WT and V190G cells (day 9 *P* = 0.003, day 20 *P* = 0.150, n = 3 biological replicates, unpaired, 2-tailed Student’s t-test), respectively. Data are presented as mean ± SD. Source data

Recent results indicate that 55LCC complex both promotes the recycling of RSL24D1 from cytoplasmic pre-60S ribosome subunits back to the nucleolus^15^ and regulates proteostasis during replication^16^. Of note, allelic variants in members of the 55LCC are associated with a strikingly similar range of neurodevelopmental defects^20–23,25,26,32^ (Fig. 2c). To begin to investigate how variants in *AIRIM* cause specific defects in human supratentorial brain development, we separately introduced the Val190Gly (V190G) and the Arg72Trp (R72W) variants into iPS cells, generated cerebral organoids from mutant and isogenic wild-type control iPS cells, and tracked organoid growth through five stages: undifferentiated iPS cells (day 0); embryoid bodies (EBs) (day 5); organoids composed mostly of epithelial neural stem cells (day 10); initiation of cortical lobe formation and neurogenesis (day 15) and initiation of cortical plate formation (day 30) (Fig. 3a–d and Extended Data Figs. 2–4). In parallel, we generated patient specific iPS cells from fibroblasts transheterozygous for the Ile466Met and Val245Glu variants in *AFG2B*^25^ and parental controls (Fig. 3a and Extended Data Figs. 2 and 3). We subjected these iPS cells to the same cerebral organoid differentiation protocol.

The *AIRIM*^*V190G*^ and *AIRIM*^*R72W*^ allelic variants did not affect proliferation or pluripotency of starting iPS cells (Extended Data Fig. 2). Similar results were obtained for *AFG2B* patient-derived iPS cells (Extended Data Fig. 2). During organoid formation, *AIRIM*^*V190G*^ and *AFG2B*^*I466M/V245E*^ mutant iPS cells differentiated into day 5 EBs and day 10 neural epithelia without obvious defects (Fig. 3c,d and Extended Data Fig. 3), whereas *AIRIM*^*R72W*^ organoids exhibited subtle reductions in size starting at day 10 (Extended Data Fig. 3c). By contrast, organoids carrying each of the allelic variants exhibited a clearly reduced size and less-elongated NE buds relative to controls on day 15 (Fig. 3c,d and Extended Data Figs. 3 and 4).

We reasoned that the observed differences in organoid size between controls and allelic variant organoids could arise from increased cell death, decreased cell proliferation or both. Staining of sectioned day 15 organoids revealed comparable levels of cell proliferation (phospho-histone 3 (pH3) and Ki67), but increased cell death (TUNEL) in mutant versus control organoids (Fig. 3e–h and Extended Data Fig. 3f–i). Notably, this phenotype did not lead to widespread degeneration, as mutant organoids continued to develop and formed cortical plate on day 30, albeit at a smaller size (Extended Data Fig. 3a,b).

### Regulation of protein synthesis during cortical development

**Fig. 4:**
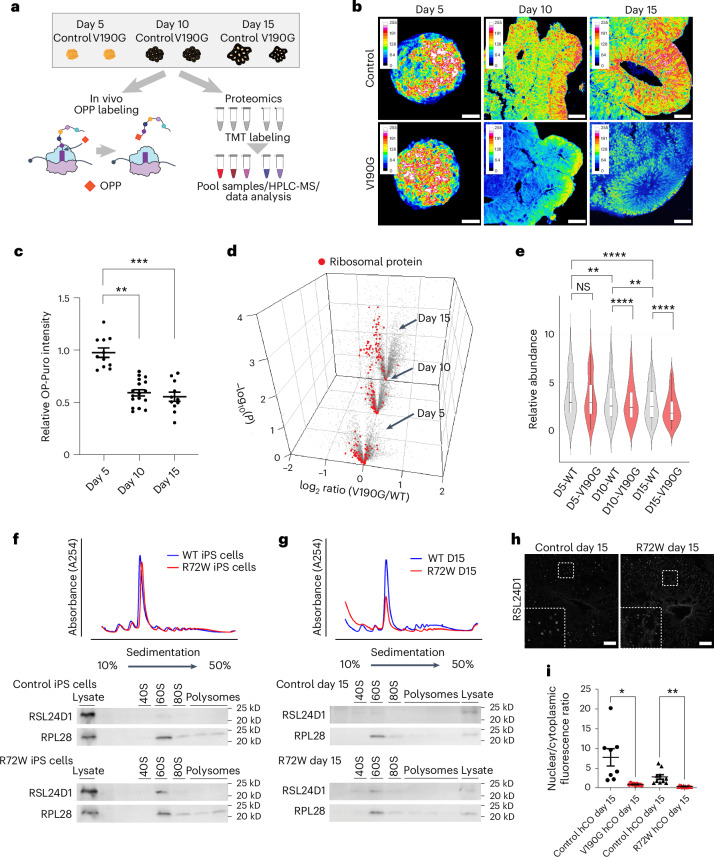
The *AIRIM*^*V190G*^ variant causes stage-specific defects in protein synthesis and ribosome levels. **a**, Experimental schemes of OP-puro based translation capacity and TMT assays. **b**, Representative images showing the OP-puro signal in control and mutant (V190G) organoids at days 5, 10 and 15. Mutant organoids exhibited lower OP-puro signals than controls starting at day 10. Scale bar, 50 μm. **c**, Quantification of OPP signal of individual NE bud at day 5, 10 and 15 mutant (V190G) organoids relative to their corresponding control. Dunn’s multiple comparisons test (two-sided). *n* = 11, 17 and 11 individual EB or NE buds from six individual organoids imaged from two independent batches. Error bars show s.e.m. Day 5 versus day 10 adjusted *P* value (*P*adj) = 8.013453 × 10^−5^; day 5 versus day 15 *P*adj = 4.692448 × 10^−5^, **P < 0.01, ***P < 0.001. **d**, Volcano plots of the −log_10_-transformed *P* value versus the log_2_-transformed ratio of mutant/control organoids at days 5, 10 and 15 (*n* = 3,539, 3,544 and 3,541 proteins, respectivel*y* *n* = 3 biologically independent samples). Discovery was determined using the two-stage linear step-up procedure of Benjamini, Krieger and Yekutieli, a false discovery rate (FDR)-controlling method for multiple comparisons (two-sided test). **e**, Violin plots showing the distribution of r-proteins as in **d** (*n* = 76 r-proteins; *n* = 3 biologically independent samples). Adjusted *P* values were calculated from Tukey’s multiple comparisons test (two-sided). **P < 0.01, ****P < 0.0001. Error bars show s.e.m. **f**, Polysome profiling with the corresponding western blots comparing the distribution of RSL24D1 and RPL28 in control and *AIRIM*^*R72W*^ iPS cells. Three independent batches were performed. **g**, Polysome profiling with corresponding western blots comparing the distribution of RSL24D1 and RPL28 in control and *AIRIM*^*R72W*^ day 15 organoids. Three independent batches were performed. **h**, IF showing the subcellular distribution of RSL24D1 in control and *AIRIM*^*R72W*^ day 15 organoids. Scale bar, 20 μm. Three independent batches were performed. **i**, Quantification of the nuclear/cytoplasm ratio of RSL24D1 immunofluorescence of control, *AIRIM*^*V190G*^ and *AIRIM*^*R72W*^ variant day 15 cerebral organoids. *P = 0.032, **P = 0.0042. Dunnett’s T3 multiple comparisons test (two-sided). Control for V190G, *n* = 8; control for V190G, *n* = 9; control for R72W, *n* = 10; control for R72W, *n* = 13 imaged regions from two independent batches. Error bars show s.e.m. Source data

To characterize how *AIRIM* variants affect global protein levels, we performed two orthogonal assays: O-propargyl puromycin (OPP) labelling and quantitative tandem mass tag mass spectrometry (TMT-MS) analysis (Fig. 4a). OPP is an analogue of the tyrosyl-tRNA mimic puromycin and can be used to pulse label nascent peptides to measure translation activity^33,34^. We performed OPP labelling on control and mutant organoids on days 5, 10 and 15 of differentiation (Fig. 4b). Mutant organoids exhibited similar levels of protein synthesis on day 5 when compared with controls. By contrast, OPP labelling in mutant organoids was reduced relative to control organoids on days 10 and 15 (Fig. 4b,c).

We next performed TMT-MS on day 5, 10 and 15 control and *AIRIM*^*V190G*^ organoids to systematically examine the abundance of ribosome components during organoid formation (Fig. 4d,e and Supplementary Table 3). During the formation of control organoids, we observed that ribosomal protein levels declined between days 5 and 15, suggesting that changes in ribosome levels are part of the normal differentiation process (Fig. 4d,e). By comparison, although *AIRIM*^*V190G*^ organoids on day 5 exhibited comparable ribosome protein levels, they showed a more dramatic reduction on days 10 and 15. These findings further suggest that *AIRIM* variants compromise ribosome formation and global protein synthesis, before growth defects first manifest during brain organoid formation.

**Extended Data Fig. 5:**
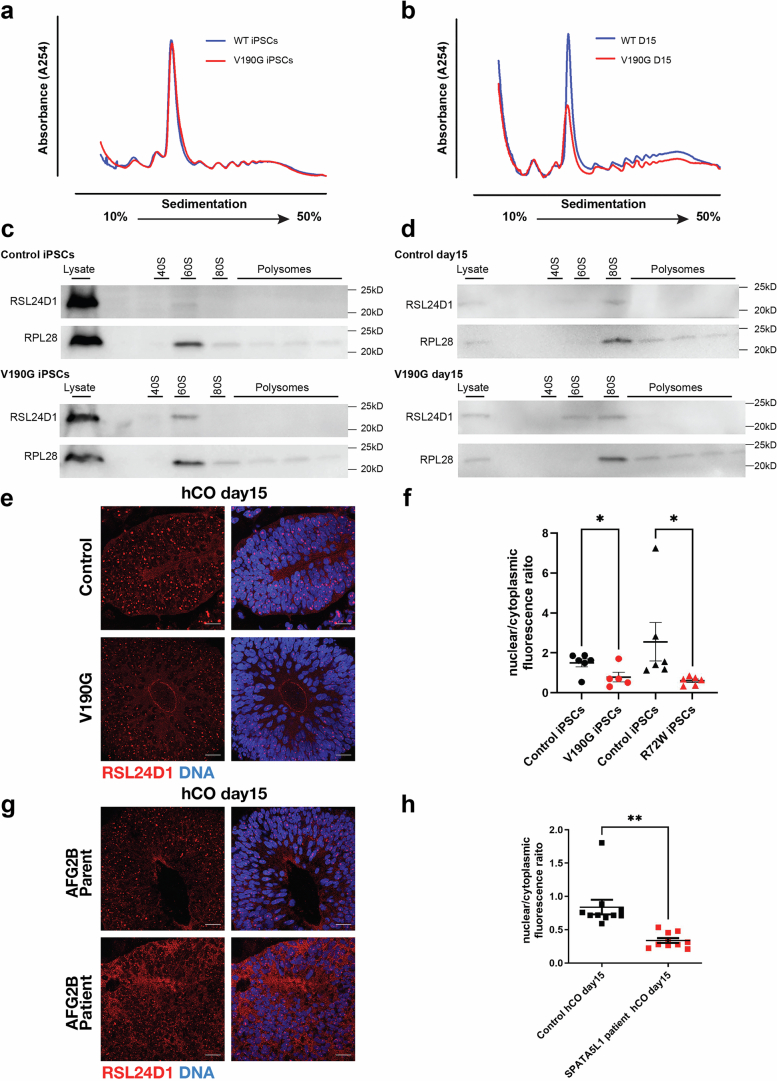
*AIRIM* variant organoids exhibit increased cytoplasmic RSL24D1. Polysome profiles of Control and *AIRIM*^*V190G*^ **a**. iPS cells and **b**. day 15 organoids. Western blots for RSL24D1 and RPL28 of the fractions corresponding to the indicated regions of the gradient for Control and *AIRIM*^*V190G*^ **c**. iPS cells and **d**. day 15 organoids. 3 independent batches were performed. **e**. IF showing the subcellular distribution of RSL24D1 in control and *AIRIM*^*R72W*^ day 15 organoids. Scale bar, 20μm. **f**. Quantification of the nuclear/cytoplasm ratio of RSL24D1 immunofluorescence of day 15 control (WT) and mutant (V190G, R72W) iPS cells. *P(V190G vs Control) = 0.0302, *P(R72W vs Control) = 0.01. Kruskal-Walli’s comparison test (two-sided). n = 6 control and 5 mutant (V190G), n = 6 control and 6 mutant (R72W) imaged regions from 2 independent batches. Error bars are SEM. **g**. IF showing the subcellular distribution of RSL24D1 in parental and AFG2B variant day 15 organoids. Scale bar, 20μm. **h**. Quantification of the nuclear/cytoplasm ratio of RSL24D1 immunofluorescence of individual neuroepithelial bud of day 15 Parental and AFG2B variant organoids. **P = 0.0013, Unpaired two-sided test with Welch’s correction. n = 10 parental and 10 AFG2B variant imaged regions from 2 independent batches. error bars are SEM. Source data

Previous studies indicated that loss of *AIRIM* or *AFG2B* results in defects in late 60S maturation, marked by disruption of RSL24D1 recycling of pre-60S subunits back to the nucleus^15^. To determine whether *AIRIM* variants found in patients exhibit similar defects, we performed sucrose gradient fractionation and assayed the distribution of RSL24D1 protein in both V190G and R72W iPS cells and day 15 organoids. While the levels of 40S, 60S, 80S and polysomes seemed similar in both control and variant iPS cells (Fig. 4f and Extended Data Fig. 5a,c), the distribution of RSL24D1 seemed slightly enriched in the 60S/80S fraction of variant iPS cells relative to controls. By contrast, the levels of both 80S monosomes and polysomes were reduced in R72W day 15 organoids, suggesting defects in ongoing mRNA translation. Furthermore, the levels of RSL24D1 associated with the 60S/80S fraction were again higher in the variant samples when compared with the control samples (Fig. 4g and Extended Data Fig. 5b,d). To further validate these results, we performed immunofluorescence (IF) analysis to examine the distribution of RSL24D1 in control and variant organoids. This analysis showed that *AIRIM* and *AFG2B* variant organoids accumulated higher levels of RSL24D1 in the cytoplasm relative to the nucleolus (Fig. 4h,i and Extended Data Fig. 5e–h). Altogether, these results indicate that AIRIM variants exhibit defects in RSL24D1 recycling from the pre-60S subunit back to the nucleolus, potentially resulting in lower levels of ribosome production and protein synthesis.

### Delayed RG differentiation in 55LCC variant organoids

**Fig. 5:**
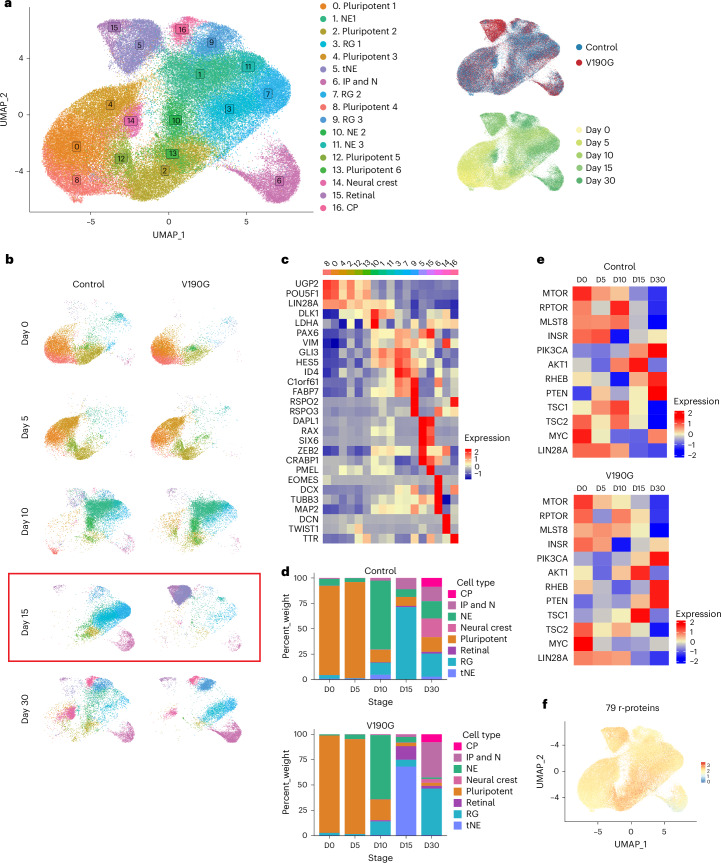
scRNA-seq reveals *AIRIM*^*V190G*^ mutant organoids display a transient delay of neuroepithelial differentiation. **a**, UMAP embedding of scRNA-seq data from one pooled sample per condition (*n* = 50 day 5 EBs, 3–6 day 10 NE, 3–6 day 15 organoids and 3 day 30 organoids per genotype, 10,000 cells were sequenced per condition). tNE, transitioning NE; IP, intermediate progenitor; N, neuron; CP, choroid plexus. **b**, Split-view of full integrated dataset, coloured by Louvain clusters. **c**, Heatmap showing the average expression of cluster marker genes related to brain development from the full integrated dataset. **d**, Quantification of different cell types identified at each time point in control and V190G samples. **e**, Heatmap showing the average expression of translation regulator genes across the development of control and V190G organoids. **f**, Gene module expression scores for a set of all 79 human r-protein genes across developmental time points.

To study how decreases in ribosome levels and mRNA translation influence organoid differentiation dynamics, we performed single-cell RNA sequencing (scRNA-seq) on control and *AIRIM*^*V190G*^ mutant organoids across five different time points (Fig. 5a,b). The sequencing data of each genotype and time point were integrated using Harmony and visualized by Uniform Manifold Approximation and Projection (UMAP) embedding^35^. These integrated data enabled a comprehensive assessment of transcriptional dynamics during organoid formation. The developmental progression and gene expression programmes of our control organoids were consistent with other studies^31,36,37^. As expected, organoid differentiation proceeded from iPS cells (POU5F1) to neural progenitors (PAX6 and vimentin (VIM)), then to intermediate progenitors (EOMES) and neurons (DCX, TUBB3 and MAP2). We also observed a small neural crest/mesenchymal population (DCN, LUM and TWIST1) as well as a population that resembles cells within the choroid plexus (TTR) (Fig. 5c).

The single-cell transcriptomes of control and *AIRIM*^*V190G*^ iPS cells and day 5 EBs largely overlapped with one another (Fig. 5a,b). Despite the observed differences in protein synthesis levels (Fig. 4b,c), the transcriptional states of NE (NE and LDHA) and transitioning NE (CDH2, VIM and ZEB2) in both control and mutant day 10 organoids were also similar (Fig. 5a–c). However, while the NE in control organoids continued to differentiate into RG progenitors (FABP7/BLBP), accompanied by signs of neurogenesis on day 15, mutant organoids exhibited delays in RG fate specification and reduced neurogenesis (Fig. 5b,d). Instead, most progenitors in day 15 *AIRIM*^*V190G*^ organoids continued to express ZEB2 (cluster 5), a regulator that functions during the NE to RG transition, indicating that while mutant NE cells initiated RG specification they did not complete this transition in a timely manner. Despite this delay, however, on day 30, both control and mutant organoids contained an abundant number of committed RGs and neurons (Fig. 5b,d).

**Extended Data Fig. 6:**
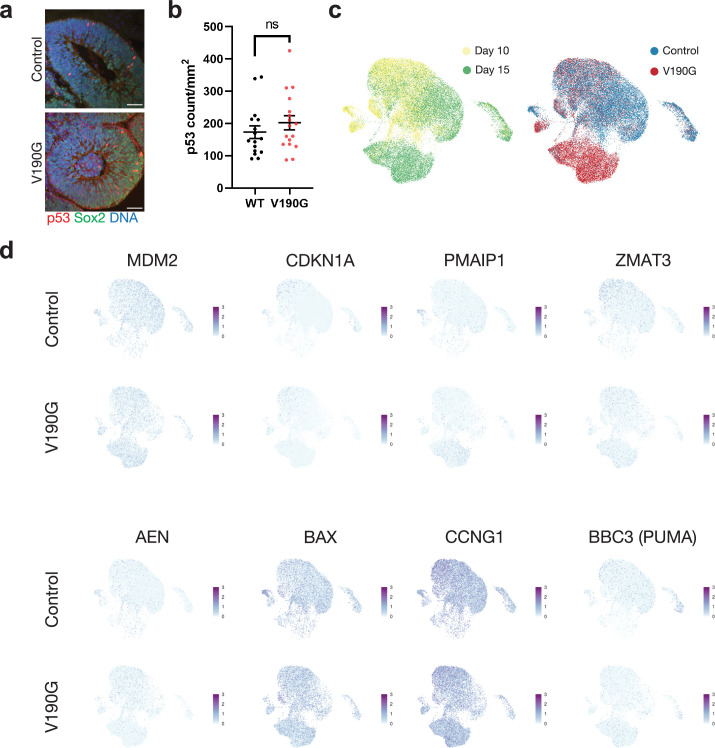
Comparison of r-protein genes and p53 target gene expression in control and AIRIM organoids. **a** Representative images showing the SOX2 (green) and p53 (red) in control (WT) and mutant (V190G) day 15 organoids. Scale bar, 50μm. **b**. Quantification of nuclear p53 signal of individual neuroepithelial bud at day 15 control (WT) and mutant (V190G) organoids. Unpaired t-test with Welch’s correction. n = 16 control and 17 mutant imaged regions from 3 independent batches, error bars are SEM. **c**. UMAP embedding of a subset of the organoid development at days 10 and 15 coloured by time point and genotype. **d**. Expression of individual p53 target genes in cells from control (WT) and mutant (V190G) samples. Source data

Of note, the expression of ribosomal protein genes and components of cell growth regulatory pathways, including the mTOR pathway, are dynamically regulated at the mRNA level during the neurodifferentiation process (Fig. 5e,f). This analysis indicated that variant organoids exhibit differences in gene expression, including increased relative expression of TSC1 at day 15 of differentiation. In addition, while many ribosomopathies result in nucleolar stress and p53 activation in other tissues^12,13^, we did not detect a dramatic increase of p53 levels or induction of a p53-dependent transcriptional response in day 15 mutant organoids relative to controls (Extended Data Fig. 6a–d). This result suggests that the pathology exhibited by variant organoids is likely caused by a p53-independent mechanism.

**Fig. 6:**
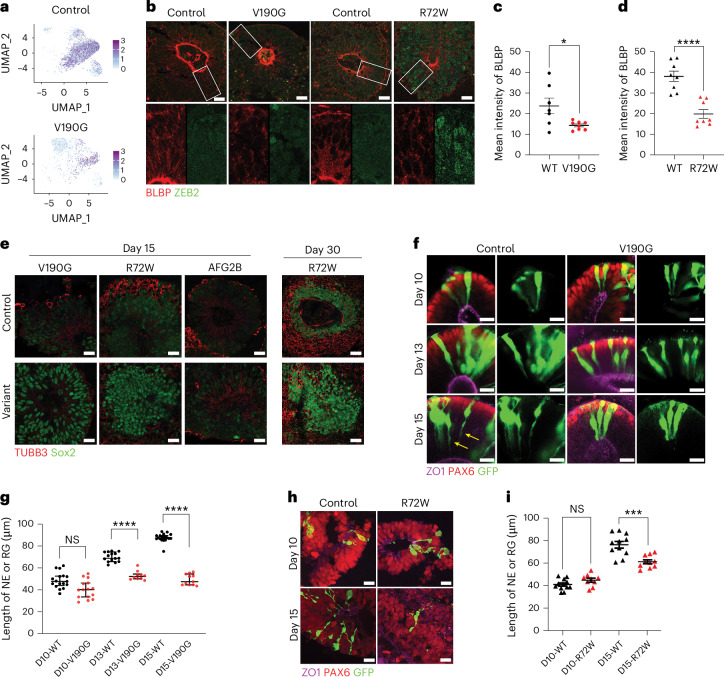
The *AIRIM*^*V190G*^ cerebral organoids exhibit impaired radial glial cell fate specification. **a**, UMAP showing FABP7 expression in control and *AIRIM* variant cells in day 15 organoids. **b**, IF image of day 15 control and mutant (V190G and R72W) organoids of transitioning NE marker ZEB2 and committed RG marker BLBP (encoded by FABP7). Note that the mutant exhibits less expression of BLBP and less-organized nuclei (marked by ZEB2). Scale bar, 20 µm. **c**, Quantification of mean BLBP IF intensity of day 15 control and mutant (V190G) organoids. *P = 0.0463. Unpaired two-tailed *t*-test with Welch’s correction. WT, *n* = 7; V190G, *n* = 8 imaged regions from two independent batches. Error bars show s.e.m. **d**, Quantification of mean BLBP IF intensity of day 15 control and mutant (R72W) organoids. ****P = 8.65486 × 10^−5^. Unpaired two-tailed *t*-test with Welch’s correction. WT, *n* = 8; R72W, *n* = 8 imaged regions from two independent batches. Error bars show s.e.m. **e**, IF image of day 15 control and *AIRIM*^*V190G*^, *AIRIM*^*R72W*^ and *AFG2B*^*I466M/V245E*^ organoids of the progenitor marker Sox2 and the neuronal differentiation marker TUBB3. Scale bar, 20 μm. Also included are IF images of day 30 control and *AIRIM*^*R72W*^ organoids stained for the progenitor marker Sox2 and the neuronal differentiation marker TUBB3. Scale bar, 40 μm. Three independent batches were performed. **f**, Representative whole-mount organoid IF images showing the morphology of neural progenitor cells (PAX6^+^), around apical (ZO1^+^) lumens, revealed by sparse labelling with GFP in control and mutant (V190G) organoids. Day 10 cells are columnar and exhibit typical NE morphology. Day 15 control cells show a thinning of apical processes (yellow arrows), whereas mutant cells still seem to be columnar. Scale bar, 20 μm. **g**, Quantification of the length of neural progenitor cells in control and mutant (V190G) organoids at days 10, 13 and 15, showing reduced length of the progenitor cells in mutant compared with control organoids. Cells with clear apical and basal labelling were used for quantification. Mann–Whitney *U*-test, ****P < 0.0001, two-tailed, multiple-comparison corrected. Day 10 control, *n* = 10 cells; day 10 V190G, *n* = 8 cells; day 13 control, *n* = 14 cells; day 13 V190G, *n* = 16 cells; day 15 control, *n* = 8 cells; and day 15 V190G, *n* = 8 cells. *P*adj = 0.0714, day 10; *P*adj = 9.177792 × 10^−7^, day 13; *P*adj = 1.098352 × 10^−7^, day 15. Error bars show s.e.m. **h**, Representative whole-mount organoid IF images showing the morphology of neural progenitor cells revealed by sparse labelling with GFP in control and mutant (R72W) organoids. Day 10 cells are columnar and exhibit typical NE morphology. Day 15 control cells show a thinning of apical processes, whereas mutant cells do not. Scale bar, 20 μm. **i**, Quantification of the length of neural progenitor cells in control and *AIRIM*^*R72W*^ organoids at days 10 and 15. Cells with clear apical and basal labelling were used for quantification. Mann–Whitney *U*-test, ***P = 0.0009, two-tailed, day 10 control, *n* = 12 cells; day 10 V190G, *n* = 9 cells; day 15 control, *n* = 11 cells; and day 15 V190G, *n* = 10 cells. Error bars show s.e.m. Source data

IF staining of control, *AIRIM*^*V190*^ and *AIRIM*^*R72W*^ day 15 organoids confirmed the reduced FABP7/BLBP expression in mutant progenitors (Fig. 6a–d). Moreover, this analysis revealed that individual rosettes continued to express ZEB2 and were less well organized in variant organoids. Cells within control day 15 organoids began to express β-tubulin III (TUBB3), a marker of the earliest stages of neuronal differentiation (Fig. 6e). By contrast, *AIRIM*^*V190G*^, *AIRIM*^*R72W*^ and *AFG2B*^*I466M/V245E*^ variant organoids continued to express relatively high levels of SOX2 and did not exhibit robust TUBB3 expression (Fig. 6e). Some TUBB3 expression is observed in day 30 *AIRIM*^*R72W*^ organoids, albeit at lower levels relative to controls, suggesting that these variant organoids can differentiate beyond these very early developmental stages (Fig. 6e). The NE to RG transition is marked by changes in cell morphology^37^. To compare cell shape in control and mutant organoids, we performed sparse green fluorescent protein (GFP) labelling, which revealed that NE cells in both control and mutant day 10 organoids seemed to be wide, columnar and relatively short (Fig. 6f). Through days 13 to 15, however, when control progenitors became more elongated, with more constricted apical processes, mutant progenitors remained morphologically similar to those in day 10 organoids (Fig. 6f,g), consistent with a delay in their RG fate commitment. Similar results were obtained with the *AIRIM*^*R72W*^ variant (Fig. 6h,i). Together, the scRNA-seq and IF data coupled with our proteomic analyses are consistent with a model whereby the *AIRIM* variants cause a temporary delay in NE to RG differentiation.

### *AIRIM* variants alter translation of specific transcripts

**Fig. 7:**
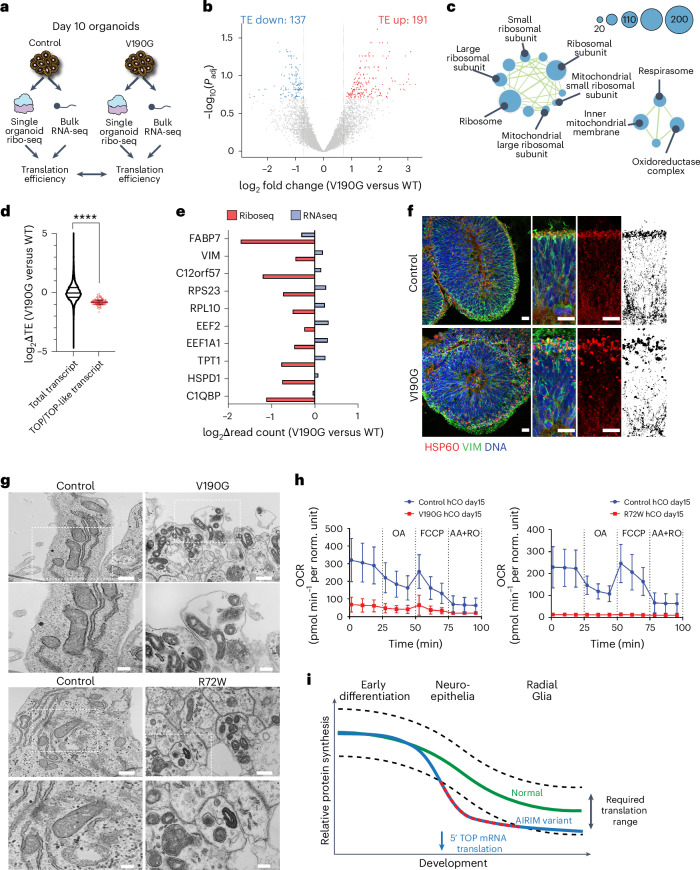
Reduced ribosome availability more profoundly affects a select subset of transcripts in differentiating neuroepithelia. **a**, Schematic of ribo-seq experiment. **b**, Volcano plot of change in TE of mutant (V190G) relative to control organoids at day 10. Red, log_2_(fold change) > 0.7 (higher TE in mutant); blue, log_2_(fold change) < −0.7 (lower TE in mutant); *n* = 2 independent batches for bulk RNA-seq, *n* = 2 control organoids and *n* = 3 mutant organoids for single-organoid ribo-seq; FDR < 0.2. Differential expression analysed by Genewise Negative Binomial GLM with quasi-likelihood tests (two-sided), adjusted for multiple comparisons using the Benjamini–Hochberg FDR method. **c**, Gene-set enrichment analysis (GO: cellular components) showing enriched terms among transcripts with decreased TE in mutant organoids. All displayed terms showed significant enrichment (FDR < 0.01) in the ‘Down’ direction from CAMERA-PR testing, indicating these cellular components are overrepresented among genes with reduced TE in mutants compared with controls. **d**, Violin plot of total transcripts and scatter-plot showing the distribution of TE changes of transcripts whose 5′ UTRs contain 5′ TOP-like motifs, shown across FDR thresholds for differential translation. ****P < 0.0001, *n* = 12,176 total and 105 TOP mRNAs. Unpaired two-sided *t*-test with Welch’s correction, *P* = 7.319627 × 10^−58^. **e**, Relative change in ribo-seq cpm (red) and RNA-seq cpm (blue) of mutant (V190G) day 10 organoids compared with controls for selected high-confidence transcripts. **f**, IF image of day 15 control and mutant (V190G) organoids of VIM (green) and mitochondrial matrix HSP60 (red). Note that the mutant exhibits more aggregated mitochondria. Scale bar, 20 μm. Three independent batch were performed. **g**, TEM micrographs of control and mutant (both V190G and R72W) day 15 organoids. This analysis shows the variant cells exhibit morphologically defective mitochondria, which are often located in autophagic vesicles. Scale bar, 500 nm; zoomed in scale bar, 200 nm. **h**, OCRs in control, *AIRIM*^*V190G*^ and *AIRIM*^*R72W*^ day 15 organoids. Control for V190G, *n* = 5; V190G, *n* = 6; control for R72W, *n* = 6; R72W, *n* = 6 biological replicates. Error bars show s.e.m. **i**, Model describing how reduced levels of ribosomes in *AIRIM* variant cerebral organoids results in decreased translation of key mRNAs encoding translation machinery components and neurogenesis factors. Error bars show s.e.m. Source data

**Extended Data Fig. 7:**
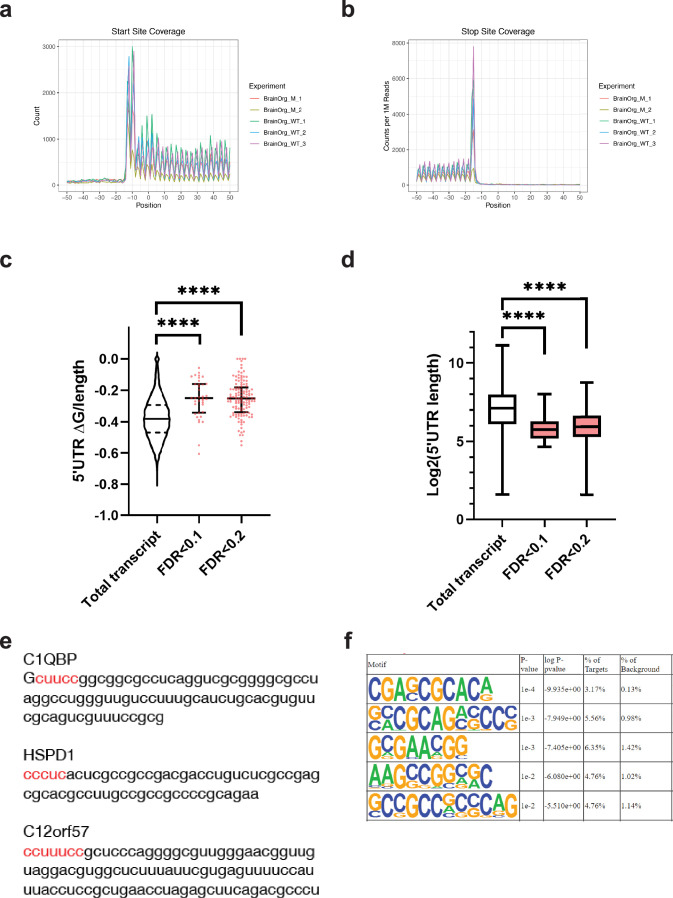
Quality control and analysis of single-organoid ribo-seq analysis. **a** The ribo-seq data exhibit high start site coverage across samples based upon metagene analysis of CDS regions. **b**. The ribo-seq data exhibit high stop site coverage across samples based upon metagene analysis of CDS regions. **c**. Violin/scatter dot plot quantifying TE changes relative to predicted 5’UTR free energy normalized to UTR length. ****P < 0.0001. Dunnett’s T3 multiple comparisons test(two-sided). n = 7982 (total), 37 (FDR < 0.1), 140 (FDR < 0.2) transcripts, *P* = 6.016844e-07(total vs FDR < 0.1), *P* = 2.003477e-19(total vs FDR < 0.2). **d**. Box dot plot quantifying TE changes relative to log2 transformed 5’UTR length. ****P < 0.0001. Dunnett’s T3 multiple comparisons test(two-sided). n = 7982 (total), 37 (FDR < 0.1), 140 (FDR < 0.2) transcripts, *P* = 9.487886e-11(total vs FDR < 0.1), *P* = 2.29069e-15 (total vs FDR < 0.2). In the order of groups “total transcript”, “FDR < 0.1”, “FDR < 0.2”, Minimum=1.58, 4.64, 1.58, Maximum=11.1, 8.03, 8.76, median=7.12, 5.75, 5.99, Q1(25%, lower bound)=6.11, 5.25, 5.29, Q3(75%, upper bound)=7.98, 6.23, 6.69, lower whisker=3.30, 4.64, 3.18, upper whisker=10.79, 7.70, 8.77,. **e**. Example of TOP-like element containing transcripts identified in this study. **f**. Motifs enriched in the 5’UTRs of *AIRIM*^*V190G*^ sensitive transcripts are shown. *P* values were calculated based on the hypergeometric distribution (two-sided, multiple-comparison corrected test). Source data

Translation and ribosome defects first arose on day 10, preceding the observed reduction in cell survival and altered cell fate commitment, as revealed by scRNA-seq (Figs. 4b–e and 5). To further dissect the mechanism underlying the temporal specificity of these phenotypes, we performed ribosome profiling via microfluidic isotachophoresis (Ribo-ITP)^38^ and assayed ribosome occupancy on mRNAs from single organoids, as well as bulk RNA-seq using day 10 organoids (Fig. 7a, Extended Data Fig. 7a,b and Supplementary Tables 4 and 5). We observed that a subset of genes important for cell survival, RG commitment, neurogenesis and early brain development^39^ exhibited dramatically lower translational efficiency (TE) in *AIRIM*^*V190G*^ organoids relative to controls, including TPT1, FABP7 and VIM (Fig. 7b–e). These genes were also enriched for ribosome components, ribosome biogenesis factors and other mRNA translation factors (Supplementary Table 6). Of note, certain mitochondrial components also exhibited reduced TE in mutant organoids (Fig. 7e).

Next, we investigated what specific mRNA features contributed to the differential sensitivity to AIRIM complex perturbations and modest reductions in ribosome availability. Previous studies show that the length and structure of 5′ untranslated regions (UTRs) impact mRNA translation^40,41^. Indeed, we found that transcripts with lower TE in mutant organoids showed shorter and less-structured 5′ UTRs relative to unaffected mRNAs (Extended Data Fig. 7c,d). In addition, many of these mRNAs contain 5′ terminal oligopyrimidine (5′ TOP) motifs, which makes them highly sensitive to fluctuations in global translation levels and ribosome availability (Fig. 7d, e)^41^. The 5′ TOP motif is found in all 79 human ribosomal protein mRNAs, as well as other mRNAs encoding factors involved in translation^42,43^. Here, TOP/TOP-like elements are found in 83 of 137 (61%) transcripts whose translation are disproportionately reduced in mutant organoids. Of note, VIM, a marker of both RG and epithelial-to-mesenchymal transition, and C12orf57, an important factor for early brain development, contain TOP-like elements within their 5′ UTRs^44–46^. Transcripts of both genes exhibit lower ribosome occupancy in *AIRIM*^*V190G*^ organoids compared with controls (Fig. 7e). In addition, transcripts encoding the mitochondrial proteins C1QBP and HSPD1 also contain TOP-like elements in their 5′ UTR and exhibited reduced translation in mutant organoids (Fig. 7e).

IF analysis of the mitochondrial matrix protein Hsp60 revealed abnormally aggregated mitochondria in day 15 mutant organoids, indicative of impaired mitochondrial function (Fig. 7f). Further examination using transmission electron microscopy (TEM) revealed significant mitochondrial morphological defects in variant organoids (Fig. 7g). Many of these abnormal mitochondria were enriched in vesicles, indicative of ongoing mitophagy, thus providing an explanation for their aggregated appearance by IF. Variant organoids also displayed dramatically reduced oxygen consumption rates (OCRs) on day 15 (Fig. 7h). Altogether, these data reveal that a subset of genes involved in promoting cell survival and neurodevelopment exhibits higher sensitivity to variation in ribosome abundance, thereby conferring vulnerability to mild perturbations in the ribosome biogenesis pathway (Fig. 7i). Moreover, we speculate that impaired mitochondrial function observed on day 15 may contribute to increased cell death in *AIRIM* variant organoids.

### Increased translation suppresses 55LCC variant phenotypes

**Fig. 8:**
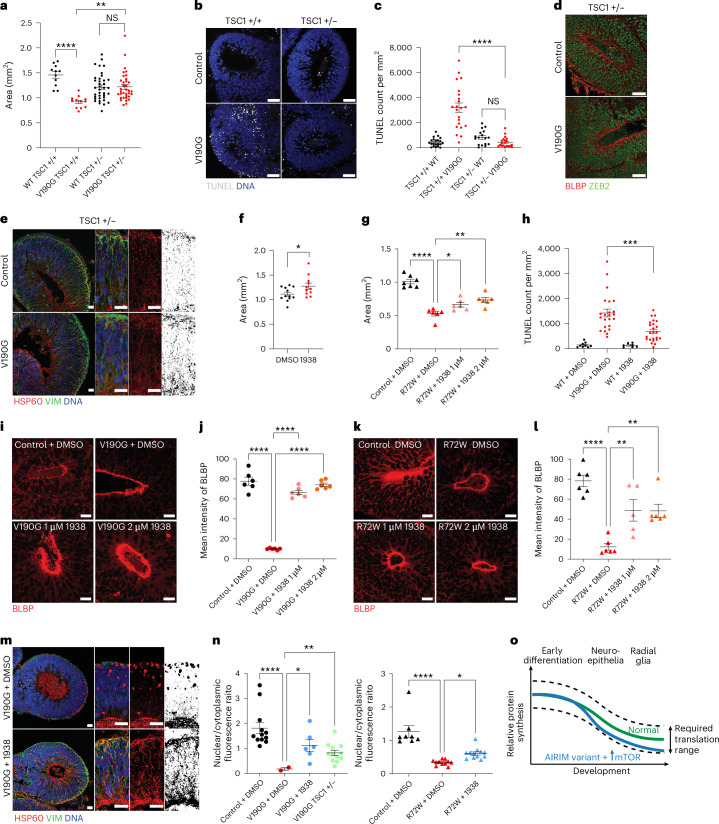
Enhancing global protein synthesis suppresses cell survival and developmental defects within *AIRIM*^*V190G*^ organoids. **a**, Quantification of bright-field images at day 15. V190G TSC1+/− neural tissue is enlarged relative to V190G TSC1+/+; **P < 0.01; ****P < 0.0001; Dunnett’s multiple comparison, unpaired two-sided *t*-test with Welch’s correction. WT TSC1+/+, *n* = 11; V190G TSC1+/+, *n* = 12; WT TSC1+/−, *n* = 37; V190G TSC1+/−, *n* = 35, from two independent batches; *P* = 1.734489 × 10^−6^ (TSC1+/+ WT versus TSC1+/+ V190G); *P* = 4.751388 × 10^−6^ (TSC1+/+ V190G versus TSC1 +/− V190G); and *P* = 0.9354378 (TSC1+/− WT versus TSC1+/− V190G). Error bars show s.e.m. **b**, Representative images showing the TUNEL signal in control TSC1+/+, V190G TSC1+/+, control TSC1+/− and V190G TSC1+/− organoids at day 15. Scale bar, 50 μm. **c**, Quantification of TUNEL signal of individual NE bud. TUNEL counts were normalized to the area of the imaged bud. ****P < 0.0001, unpaired, two-sided *t*-test with Welch’s correction. WT TSC1+/+, *n* = 21; V190G TSC1+/+, *n* = 22; WT TSC1+/−, *n* = 17; V190G TSC1+/−, *n* = 20 NE buds from two independent batches; *P* = 2.310684 × 10^−7^ (TSC1+/+ V190G versus TSC1+/− V190G); and *P* = 0.1644 (TSC1+/− WT versus TSC1 +/− V190G). Error bars show s.e.m. **d**, IF image of day 15 control (WT TSC1+/−) and mutant (V190G TSC1+/−) organoids of transitioning NE marker ZEB2 and committed RG marker BLBP. Scale bar, 50 μm. Two independent batch were performed. **e**, IF image of day 15 control (WT TSC1+/−) and mutant (V190G TSC1+/−) organoids of VIM (green) and mitochondrial matrix HSP60 (red). Scale bar, 20 μm. Two independent batch were performed. **f**, Quantification of size of day 15 mutant (V190G) organoids treated with vehicle (DMSO) or 1 μM PI3Kα activator UCL-TRO-1938. *P < 0.05. Unpaired two-sided *t*-test with Welch’s correction. WT + DMSO, *n* = 7; V190G + DMSO, *n* = 12; V190G + 1938 1 μM, *n* = 11, organoids from two independent batches; *P* = 0.037447. Error bars show s.e.m. **g**, Quantification of size of day 15 mutant (R72W) organoids treated with vehicle (DMSO) or 1 μM or 2 µM PI3Kα activator UCL-TRO-1938. *P = 0.0373, **P = 0.0015, ****P = 8.67138 × 10^−7^. Dunnett’s multiple comparison, unpaired *t*-test with Welch’s correction (two-sided). Control + DMSO, *n* = 7; R72W + DMSO, *n* = 6; V190G + 1938 1 μM, *n* = 6; V190G + 1938 2 μM, *n* = 6 organoids from two independent batches. Error bars show s.e.m. **h**, Quantification of TUNEL signal of individual NE bud. TUNEL counts were normalized to the area of the imaged bud. ***P < 0.001, Dunnett’s T3 multiple comparisons test (two-sided). WT + DMSO, *n* = 9; V190G + DMSO, *n* = 11; WT + 1938, *n* = 7; V190G + 1938, *n* = 13 from two independent batches. *P* = 0.0008 (V190G + DMSO versus V190G + 1938). Error bars show s.e.m. **i**, Representative IF images of day 15 control and mutant (V190G, 1938 1 μM and 1938 2 μM) organoids of BLBP (red). Scale bar, 20 μm. **j**, Quantification of mean BLBP IF intensity of day 15 control and mutant (V190G, 1938 1 μM and 1938 2 μM) organoids. ****V190G versus control, *P* = 1.60703 × 10^−5^; ****V190G versus 1938 1 μM, *P* = 1.39136 × 10^−6^; ****V190G versus 1938 2 μM, *P* = 2.35979 × 10^−7^. Dunnett’s multiple comparison unpaired *t*-test with Welch’s correction (two-sided). Control + DMSO, *n* = 6; V190G + DMSO, *n* = 6; control + 1938, *n* = 6; V190G + 1938, *n* = 6 imaged regions from two independent batches, error bars are s.e.m. **k**, Representative IF images of day 15 control and mutant (R72W, 1938 1 μM and 1938 2 μM) organoids of BLBP (red). Scale bar, 20 μm. **l**, Quantification of mean BLBP immunofluorescence intensity of day 15 control and mutant (R72W, 1938 1 μM and 1938 2 μM) organoids. **R72W versus 1938 1 μM, *P* = 0.0027; **R72W versus 1938 2 μM, *P* = 0.0037; ****R72W versus control, *P* = 1.49216 × 10^−6^. Dunnett’s multiple comparison, unpaired two-sided *t*-test with Welch’s correction. Control + DMSO, *n* = 6; R72W + DMSO, *n* = 6; R72W + 1938, *n* = 5; R72W + 1938, *n* = 6 imaged regions from two independent batches. Error bars show s.e.m. **m**, IF images of day 15 control (V190G and DMSO 0.1%) and treated (V190G, UCL-TRO-1938 1 μM) organoids stained for VIM (green) and mitochondrial matrix HSP60 (red). Scale bar, 20 μm. **n**, Quantification of the nuclear/ cytoplasm ratio of RSL24D1 IF of control + DMSO, *AIRIM*^*V190G*^ + DMSO, *AIRIM*^*V190G*^ + 1938 2 μM, *AIRIM*^*V190G*^ TSC1+/− and control + DMSO, *AIRIM*^*R72W*^ + DMSO, *AIRIM*^*R72W*^ + 1938 2 μM day 15 cerebral organoids. ****AIRIMV190G versus control, *P* = 1.26963 × 10^−5^; *AIRIM^*V190G*^ versus 1938 2 μM, *P* = 0.024; **AIRIM^*V190G*^ versus *AIRIM*^*V190G*^ *TSC1*+/−, *P* = 0.0018; ****AIRIM^*R72W*^ versus control, *P* = 1.0138 × 10^−5^; *AIRIM^*R72W*^ versus 1938 2 μM, *P* = 0.0491. Dunnett’s multiple comparison, unpaired two-sided *t*-test with Welch’s correction. Control (V190G + DMSO), *n* = 12; V190G + DMSO, *n* = 2; V190G + 1938 2 μM, *n* = 6; V190G TSC1+/−, *n* = 12; control (R72W + DMSO), *n* = 8; R72W + DMSO, *n* = 11; V190G + 1938 2 μM, *n* = 12 organoids from two independent batches. Error bars show s.e.m. **o**, Model describing a potential explanation for the observed suppression of *AIRIM*^*V190G*^ phenotypes by UCL-TRO-1938. Source data

**Extended Data Fig. 8:**
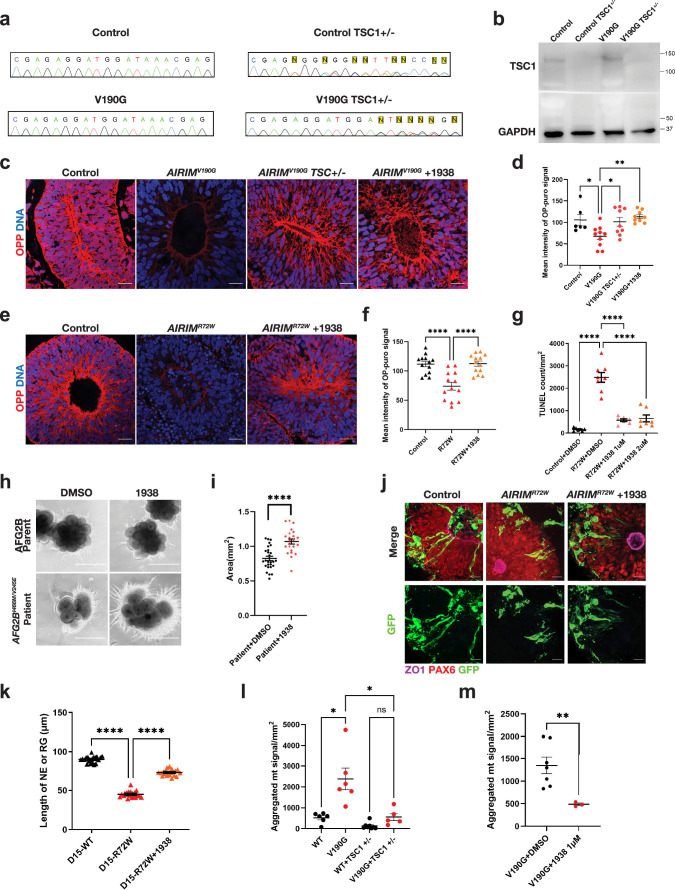
Pharmacological activation of PI3Kα suppresses cerebral organoid growth defects caused by *AIRIM* and *AFG2B* variants. **a** Sanger sequencing of control and *AIRIM*^*V190G*^ *TSC1* mutant clones. **b**. Western blot showing TSC1 expression in control and *TSC1* mutant clones. 3 independent batches were performed. **c**. Representative images showing the OP-puro signal in day 15 control, *AIRIM*^*V190G*^, *AIRIM*^*V190G*^ TSC +/-, and *AIRIM*^*V190G*^ organoids treated with 2 µM UCL-TRO-1938. Scale bar, 20 μm. **d**. Quantification of mean OP-puro signal intensity of day 15 control, *AIRIM*^*V190G*^, *AIRIM*^*V190G*^ *TSC1 +/-*, and *AIRIM*^*V190G*^ organoids treated with 2 µM UCL-TRO-1938. *P(*AIRIM*^*V190G*^ vs Control) = 0.0194, *P(*AIRIM*^*V190G*^ vs *AIRIM*^*V190G*^ *TSC1* +/-) = 0.0188, **P(*AIRIM*^*V190G*^ vs *AIRIM*^*V190G*^ + 1938) = 0.0014. Dunnett’s multiple comparison Unpaired two-sided t-test with Welch’s correction (two-sided). n (Control) = 6, n (*AIRIM*^*V190G*^) = 10, n (*AIRIM*^*V190G*^ *TSC1 +/-*) = 9, n (*AIRIM*^*V190G*^ + 1938) = 9 imaged regions from 2 independent batches, error bars are SEM. **e**. Representative images showing the OP-puro signal in day 15 control, *AIRIM*^*R72W*^, and *AIRIM*^*R72W*^ organoids treated with 2 µM UCL-TRO-1938. Scale bar, 20 μm. **f**. Quantification of mean OP-puro signal intensity of day 15 control, *AIRIM*^*R72W*^, and *AIRIM*^*R72W*^ organoids treated with 2 µM UCL-TRO-1938. ****P(*AIRIM*^*R72W*^ vs Control) = 9.59586e-05, ****P(*AIRIM*^*R72W*^ vs *AIRIM*^*R72W*^ + 1938) = 7.00537e-05. Dunnett’s multiple comparison Unpaired two-sided t-test with Welch’s correction. n (Control) = 13, n (*AIRIM*^*R72W*^) = 13, n (*AIRIM*^*R72W*^ + 1938) = 13 imaged regions from 2 independent batches, error bars are SEM. **g**. Quantification of TUNEL signal of individual neuroepithelial buds. TUNEL counts were normalized to the area of the imaged bud. ****P(*AIRIM*^*R72W*^ vs Control) = 2.43186e-07, ****P(*AIRIM*^*R72W*^ vs *AIRIM*^*R72W*^ + 1938 1 µM) = 2.8307e-06, ****P(*AIRIM*^*R72W*^ vs *AIRIM*^*R72W*^ + 1938 2 µM) = 1.73774e-05. Dunette’s multiple comparison Unpaired t-test with Welch’s correction (two-sided). n (Control+DMSO) = 7, n (R72W + DMSO) = 8, n (R72W + 1938 1 µM) = 7, n (R72W + 1938 2 µM) = 7 imaged regions from 2 independent batches, error bars are SEM. **h**. Bright-field images of day 15 control (parent) and patient (*AFG2B*^*I466M/V245E*^) organoids treated with vehicle (DMSO) or 2 μM PI3Kα activator UCL-TRO-1938 (1938). Scale bar, 1 mm. **i**. Quantification of size of day 15 *AFG2B*^*I466M/V245E*^ patient organoids treated with vehicle (DMSO) or 2 μM PI3Kα activator UCL-TRO-1938(1938). ****P<0.0001. Unpaired two-sided t-test with Welch’s correction. n = 28 DMSO and 23 UCL-TRO-1938 treated organoids, *P* = 6.2856e-06, error bars are SEM. **j**. Representative whole mount organoid immunofluorescence images showing the morphology of neural progenitor cells revealed by sparse labelling with GFP in control, *AIRIM*^*R72W*^ organoids, and *AIRIM*^*R72W*^ organoids treated with 2 µM UCL-TRO-1938 (1938). Scale bar, 20 µm. **k**. Quantification of the length of neural progenitor cells in day 15 control, *AIRIM*^*R72W*^ organoids, and *AIRIM*^*R72W*^organoids treated with 2 µM UCL-TRO-1938 (1938). Cells with clear apical and basal labelling were used for quantification. ****P(*AIRIM*^*R72W*^ vs Control) = 8.35763e-21, ****P(*AIRIM*^*R72W*^ vs AIRIM^*R72W*^ + 1938 2 µM) = 1.84064e-15. Dunette’s multiple comparison Unpaired t-test with Welch’s correction (two-sided). n (control) = 20 cells, n (*AIRIM*^*R72W*^) = 20 cells, and n (*AIRIM*^*R72W*^ + 1938 2 µM) = 20 cells. Error bars are SEM. **l**. Quantification of aggregated mitochondrial signal of individual neuroepithelial bud at day 15 organoids. *P < 0. 05; Dunnett’s multiple comparison Unpaired two-sided t-test with Welch’s correction. n(WT) = 6, n(V190G) = 6, n(WT + TSC1 +/-) = 8, n(V190G + TSC1 +/-) = 5 imaged regions from 2 independent batches, *P* = 0.0002(WT vs V190G), 0.5622(WT vs WT TSC1 +/-), 0.9995(WT vs V190G TSC1 +/-). error bars are SEM. **m**. Quantification of aggregated mitochondria signal of individual neuroepithelial bud at day 15 V190G and V190G + 1938 1 μM organoids. **P < 0.01. Unpaired two-sided t-test with Welch’s correction. n (V190G + DMSO) = 7, n (V190G + 1938 1 μM) = 3 imaged regions from 2 independent batches, *P* = 0.002938, error bars are SEM. Source data

mTOR signalling controls global protein synthesis levels by phosphorylating key translation factors including eIF4E binding proteins (4E-BPs), resulting in upregulation of cap-dependent translation^47^. mTORC1 is negatively regulated by the TSC1/2 complex^47^. scRNA-seq revealed that expression of mTOR and other genes that promote global protein synthesis, including Insulin Receptor (INSR) and Lin28, decreased during early NE development in human organoids (Fig. 5e). Variant organoids exhibited increased TSC1 expression, consistent with OPP labelling and TMT analysis (Fig. 5e). Given that the translation of 5′ TOP-element containing mRNAs is highly sensitive to mTOR activity^41^, we next tested whether increasing mTOR signalling using either genetic or pharmacological approaches could rescue defects associated with the *AIRIM*^*V190G*^ variant. We hyperactivated mTORC1 by generating heterozygous *TSC1* knockout control and mutant iPS cells and used them to generate brain organoids (Fig. 8a–e and Extended Data Fig. 8a,b). *TSC1* haploinsufficiency increased global protein synthesis and suppressed the phenotypes associated with *AIRIM*^*V190G*^ mutant organoids, including the organoid growth defects, increased cell death, delayed RG commitment and aberrant mitochondrial morphology (Fig. 8a–e and Extended Data Fig. 8c,d,l).

Recent efforts have identified a chemical agonist of PI3Kα, referred to as UCL-TRO-1938, that can be used to increase mTORC1 signalling^48^. We treated *AIRIM*^*V190G*^ and *AIRIM*^*R72W*^ organoids with 1 µM or 2 µM of UCL-TRO-1938 starting at day 10 of the differentiation protocol. Similar to *TSC1* loss-of-function, treatment with UCL-TRO-1938 resulted in increased protein synthesis in organoids carrying allelic variants, as marked by increased OPP labelling (Extended Data Fig. 8e,f). We assayed organoids on day 15 and observed that addition of the compound also alleviated the developmental defects associated with the *AIRIM*^*V190G*^, *AIRIM*^*R72W*^ and *AFG2B*^*I466M/V245E*^ variants, to varying degrees (Fig. 8f–m and Extended Data Fig. 8g–l). Moreover, UCL-TRO-1938 treatment partially suppressed RSL24D1 trafficking defects in AIRIM variant organoids (Fig. 8n), suggesting that pharmacologically increasing protein synthesis may be able to overcome minor decreases in ribosome availability. Together, these results demonstrate that elevating mTOR signalling counteracts the growth defects and cell fate specification delays associated with AIRIM complex variants in cerebral organoids (Fig. 8o).

## Discussion

**Extended Data Fig. 9:**
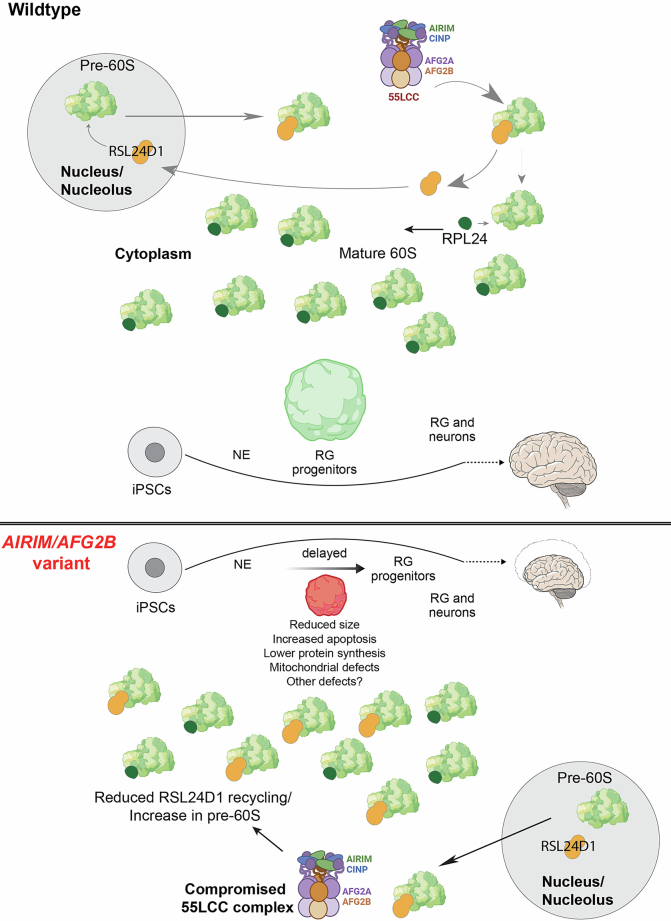
Model for how *AIRIM* and *AFG2B* variants cause differentiation defects in human cerebral organoids. (Top) The 55LCC complex promotes the maturation of the pre-60S subunit by fostering RSL24D1 recycling. Normal levels of mature 60S subunits allow for the normal differentiation of cerebral organoids. (Bottom) Allelic variants in *AIRIM* and *AFG2B* perturb the function of the 55LCC complex, compromising the maturation of pre-60S subunits. This results in reduced protein synthesis capacity in cerebral organoids, marked by delayed differentiation, reduced size, increased apoptosis, and mitochondrial defects.

Collectively, our data support a model whereby the dynamic regulation of global mRNA translation plays an important role in early brain development. Leveraging iPS cell-derived cerebral organoids, our study identifies a ribosome bottleneck of early human brain development during which ribosome numbers decline but global protein synthesis must be maintained above a minimum threshold to support cell survival and cell fate specification. Previous studies have shown the importance of the NE to RG cell transition in regulating brain size across different primate species^37^. Stage-specific variation in ribosome availability may render differentiating cells sensitive to perturbations in ribosome biogenesis (Extended Data Fig. 9). Similar changes in mRNA translation levels and ribosome availability have been observed in the developing mouse brain^49^. Moreover, recent findings reveal that alterations in rRNA modification can influence neural cell fate^50^. Disruptions to ribosome biogenesis and mRNA translation are linked with other neurodevelopmental disorders, including Fragile X Syndrome^51^, those caused by Nlgn3 loss-of-function^52^ and autism^53^. These studies, together with data presented here, highlight the importance of the dynamic regulation of ribosome levels and function during different stages of neurodevelopment.

Here, we identify seven pathogenic *AIRIM* allelic variants across 11 different families that are tightly associated with a range of neurodevelopmental disorders. These phenotypes are similar to those associated with alleles in other components of the AIRIM complex, including *AFG2A*, *AFG2B* and *CINP*^20–23,25,26,32^, and are predominantly associated with global developmental delay, intellectual disability, microcephaly, infantile seizures, muscular hypotonia, limb spasticity, dystonia and feeding problems, as well as hearing and vision impairment (Fig. 2d provides a comparative summary). Notably, patients who carry *AFG2A* and *AFG2B* allelic variants exhibit similar neuroimaging features^20,23–25^. It is worth noting that the severity of the imaging findings, particularly the extensive atrophy, seems slightly more pronounced in patients with *AIRIM* compared with those described for *AFG2A*. Certain pathogenic variants, such as those in *UBTF*, can result in abnormal ribosomal RNA (rRNA) expression, leading to severe neuro-regression and a similar neuroimaging phenotype characterized by prominent supratentorial brain atrophy with thinning corpus callosum, abnormal myelination, with relative sparing of the cerebellum and brainstem^54^. Deleterious variants in tRNA production machinery have also been associated with NDDs^55^. Perhaps these defects converge on a common mechanism, such as inappropriate declines in mRNA translation during specific stages of brain development when ribosome availability or activity may be closer to a lower limit of a functional threshold.

Structural modelling of AIRIM variants, coupled with analyses of the available 55LCC complex structure^16^, revealed that AIRIM mutations largely decrease its interactions with other components of the 55LCC complex. It is also possible that a combination of conformational changes in AIRIM, together with reduced complex association and/or stability, affect 55LCC interactions with its substrates, enzyme activity or function. Of note, our data indicate that a subset of mutants strengthen AIRIM binding to CINP, while reducing or abolishing AIRIM association with AFG2A and/or AFG2B. These observations suggest that disease-related AIRIM variants not only cause destabilization of 55LCC but may also induce imbalances between the AIRIM–CINP subcomplex relative to the AFG2A–AFG2B ATPase units.

We find that allelic variants in both *AIRIM* and *AFG2B* result in widespread cell death and delays in RG specification during cerebral organoid formation. We speculate that cells undergoing the NE to RG transition need to maintain a minimum threshold of protein synthesis capacity to ensure timely differentiation. Unexpectedly, *AIRIM* and *AFG2B* variants did not cause obvious widespread activation of a p53-dependent stress response. Consistent with our findings, cells from patients carrying variants in other components of the AIRIM complex exhibit mitochondria defects^20^. Loss of the AIRIM–55LCC complex was also recently shown to disrupt proteostasis at the replisome^16^, resulting in replication stress and mitotic errors^32,56,57^. Future work will be needed to test the extent to which mitochondrial dysfunction or DNA damage contributes to the neurodevelopmental disorders associated with allelic variants in this complex. Thus, our results may provide a unified molecular framework that potentially links defects in cytoplasmic ribosomes with previously observed abnormalities in mitochondria or DNA replication in patients that carry *AFG2A/SPATA5* allelic variants^24^.

Our study further reveals that reductions in protein synthesis capacity affect the translation of specific mRNAs in developing brain organoids. Most of these mRNAs contain 5′ TOP elements. Recent work has found that transcriptional start sites in individual genes can vary across tissues, resulting in the cell-type specific inclusion of 5′ TOP and TOP-like elements with the 5′ UTRs of mRNAs^58^. Our results indicate that perturbations in 60S maturation become exacerbated during neurodifferentiation through reductions in the translation of mRNAs involved in protein synthesis. Increasing mTOR activity partially suppresses the *AIRIM* variant phenotypes, further linking the abnormalities observed in patients with defects in the regulation of protein synthesis. However, ribosome and global protein synthesis levels must be finely tuned during different developmental events. Inappropriate increases in global protein production can also have deleterious effects on brain development and function. For example, variants that disrupt the TSC1/2 complex result in noncancerous tumour growth in human brain^47^. Therefore, ribosome availability and protein synthesis will need to be adjusted in space and time to successfully treat abnormalities caused by loss-of-function alleles in ribosome assembly machinery. These results encourage future investigation into therapeutic approaches aimed at modulating protein synthesis in a cell-type and temporally specific manner.

## Methods

### Ethics statement

Ethical oversight for all human data described in this study was provided by Phoenix Children’s Hospital (Institutional Review Board no. 15-080), University College London Queen Square Institute of Neurology (22/NE/0080, project ID 310045), Heidelberg University (S-186/2012), King Faisal Hospital Specialist & Research Centre (20DG1533: RAC no. 2121053 and 23DG0161: RAC no. 2210029), National and Kapodistrian University of Athens (16434/25-07-22) and King Abdullah International Medical Research Center (Institutional Review Board 1470/24, project no. NRC23R/177/02). All human sequencing data were obtained with informed consent from patient family members. The donors received no compensation. We describe 11 families with allelic variants in *AIRIM*. Sex was assigned and reported in the appropriate figures and tables. This is not a population study, therefore sex- and gender-based analyses were not performed. Fibroblasts from an *AFG2B* patient were obtained with informed consent by Phoenix Children’s Hospital, which also allowed for their reprogramming (performed at UT Southwestern). The UT Southwestern Stem Cell Research Oversight committee provided oversight for all experiments involving iPS cells and cerebral organoids. All experiments were conducted in compliance to the principles laid out in the International Society for Stem Cell Research guidelines.

### Patient recruitment and sequencing

Patients were recruited as part of large-scale sequencing screens as previously described^25^. Exome sequencing was performed in a research setting or in accredited molecular diagnostic laboratories. Sanger sequencing was used for variant validation and segregation analysis. Variants were mapped to the predicted structure of AIRIM^59,60^.

### MRI analysis of patients

Given the diverse range of centres contributing to these patient’s cohort, there was considerable variation in magnetic resonance scanner manufacturers, sequences obtained and imaging parameters. The minimum required magnetic resonance imaging (MRI) sequences for inclusion consisted of axial T1-weighted images (T1WI) and axial T2-weighted images (T2WI), each with a section thickness of ≤5 mm. Additionally, when available, other sequences, including T2-FLAIR, susceptibility-weighted imaging, diffusion-weighted imaging/diffusion tensor imaging (DWI/DTI) and gradient-recalled echo were also reviewed when available.

### Stem cell culture and proliferation assay

Five human iPS cell lines (SCVI274, F856/parent#1, F856/parent#2, F740/proband#1, F740/proband#2) were used in this study. SCVI274 were obtained from Stanford SCVI BioBank. F856#1, 2 and F740#1, 2 were reprogrammed in this study. All iPS cell lines were cultured in mTESR-plus medium (Stem Cell Technologies, 100-0276) on Matrigel-coated plates (Corning, 354277). Cells were passaged every 3–5 days after dissociation with ReLeSR (Stem Cell Technologies, 100-0483). The medium was supplemented with Rho-associated protein kinase (ROCK) inhibitor Y-27632 (Tocris, 1254) at a final concentration of 10 µM for the first 24 h after passaging. Routine *Mycoplasma* tests were performed with Universal Mycoplasma Detection kit (ATCC, 30-1012K). For proliferation assay, cells were dissociated with ACCUTASE (Stem Cell Technologies, 07920) and were passaged onto Matrigel-coated 12-well plates at 1.0 × 10^5^ cells per well in mTESR-plus medium with 10 µM ROCK inhibitor. At 24 h after passage, medium was changed with mTESR-plus without ROCK inhibitor. Medium was then changed every 24 h. Cells were dissociated with TrypLE Express Enzyme (Thermo Fisher, 12605010) and were counted by a haemocytometer.

### Reprogramming of fibroblasts

The fibroblasts were reprogrammed using episomal vectors as previously described^61^. In brief, 1 × 10^6^ fibroblasts were electroporated with 2.5 µg of each of the following vectors: pCXLE–EGFP, pCXLE–hOCT3/4-shp53-F, pCXLE–hSK and pCXLE–hUL (Addgene, #27082, #27077, #27078 and #27080, respectively) using a NEPA21 Type II Super Electroporator (Bulldog-Bio) using the manufacturer’s recommended parameters. After electroporation, fibroblasts were collected and transferred equally to a six-well plate coated with Matrigel in DMEM (Sigma) supplemented with 10% fetal bovine serum. After 48 h, medium was replaced with mTESR-plus medium. The medium was refreshed every 24 h from then on. After 3–5 weeks, tentative human iPS cell colonies were manually isolated and expanded for analysis.

### CRISPR-mediated gene editing and knock out

For the SCVI274 AIRIM mutant line, 2 × 10^6^ parental cells were resuspended in 100 µl OPTI-MEM with 5 µg of px458-sgRNA plasmid and 5 µg of ssDNA donor. The cells were then electroporated at poring pulse 150 V/length 2.5 ms/interval 50 ms, transfer pulse 20 V/length 50 ms/interval 50 ms. At 48 h after electroporation, cells were sorted for GFP positivity on a FACS-Aria sorter. The sorted cells were plated onto Matrigel-coated six-well plates at 2,000 cells per well in mTESR-Plus with 10 µM ROCK inhibitor. At 7–14 days after plating, colonies were plated onto a 24-well plate. Colonies were screened by PCR and Sanger sequencing. Homozygous mutant clones were expanded, and genotypes were reconfirmed after expansion. For the TSC1 knock out, 2 × 10^6^ parental cells were resuspended in 100 µl OPTI-MEM with 5 µg of px458-sgRNA plasmid. The cells were electroporated, sorted and screened as described above. Sequences of sgRNA, donor and PCR primers are available in Supplementary Table 7.

### Teratoma assay

Female immunodeficiency NOD-SCID mice (~10 weeks old) were used for teratoma assays with UT Southwestern Institutional Animal Care and Use Committee approval (APN-2018-102430). Mice were housed in a 12-h light–dark cycle, 22.1–22.3 °C and 33–44% humidity. Human iPS cells were dissociated into a single-cell suspension, and then resuspended at a concentration of 1 × 10^7^ cells per ml in a mixture of 50% Matrigel and 50% culture medium containing ROCK inhibitor. Cells were injected subcutaneously into the flanks of female NOD/SCID immunodeficient mice with a total of 1 × 10^6^ cells per injection site. Teratomas were isolated after 8 weeks of growth once visible tumours had formed, and the tissue was fixed in 4% PFA for 48 h. Tumour samples were submitted to the UT Southwestern Histopathology Core facility for paraffin embedding, sectioning and haematoxylin and eosin (H&E) staining. The germ layer contribution was determined through imaging and histological examination of H&E-stained tumour sections.

### Generating cerebral organoids

Cerebral organoids were generated using Stemdiff Cerebral Organoid kit (Stem Cell Technologies, 08570). The timing of the kit protocol (Stem Cell Technologies Document, DX21849) was followed. The EB formation step was modified to generate organoids with enhanced telencephalic identity^37^. In Aggrewell 800 plates (Stem Cell Technologies, 34815), 6 × 10^5^ cells in 2 ml EB formation medium were added per well to achieve 2,000 cells per EB. During the EB formation period, the medium was changed by 50% every day with an electronic auto dispenser at the slowest pipetting speed. At neural induction, Aggrewell were changed with induction medium three times to achieve <0.5% remaining EB formation medium. Matrigel embedding for individual organoids has been previously described^28,29^. Matrigel embedding for large-scale assay (>100 organoids per batch) was performed by following the previously described protocol^37^. All comparisons between samples and treatments were performed on organoids generated using identical protocols. For the sparse labelling of neural progenitor cells, 5 ml CytoTune emGFP Sendai fluorescence reporter (Thermo Fisher, A16519) was added to Aggrewells when the organoids were switched to neural induction medium.

Brain organoid was generated from H9 human embryonic stem cells as previously reported. In brief, H9 cells were dissociated to single cells by incubating with TrypLE (Thermo Fisher) at 37 °C for 3 min. Cells were centrifuged and resuspended in stem cell culture medium containing DMEM/F12 (Corning), 20% knockout serum (Thermo Fisher), GlutaMAX (Thermo Fisher, 1:100 dilution), NEAA (Thermo Fisher, 1:100 dilution) and 50 μM β-mercaptoethanol (Thermo Fisher), supplemented with 10 ng ml^−1^ FGF2 (PeproTech) and 20 μM Y-27632 (Tocris). Then cell suspensions were plated on 96-well, round-bottom, ultra-low attachment plates (Corning) at density of 2,500 or 9,000 cells per well and incubated at 37 °C for 48 h. On day 2, 3.3 μM XAV939 (Selleck Chemicals), 250 ng ml^−1^ NOGGIN (R&D Systems) and 5 μM SB431542 (Sigma) were added to the medium and the medium was changed every 2 days. On day 6, the spheroids were moved to neural medium containing Neurobasal A (Thermo Fisher), GlutaMAX, B27 without vitamin A (Thermo Fisher), supplemented with 20 ng ml^−1^ FGF2 and 20 ng ml^−1^ EGF (PeproTech) and the medium was changed every 2 days.

### Organoid size analysis

Images of organoids in culture were taken with an inverted microscope (ECHO R4) at ×4 magnification objective. Area values were obtained by quantifying individual organoids on ImageJ, which measured area in mm^2^.

### Immunofluorescence

For cryosectioning, organoids were washed in PBS before fixing in 4% PFA at 4 °C overnight. After fixation, organoids were washed three times with PBS and were then incubated in PBS/15% sucrose for 6 h at 4 °C, and then PBS/30% sucrose for 16–24 h at 4 °C. Organoids were transferred to plastic cryomolds and were embedded in OCT compound for snap-freezing on dry-ice and were cryosectioned at a thickness of 20 µm. Sections were warmed at room temperature (RT) for 10 min and rinsed with TBS three times with 10 min each before incubating with blocking solution (0.3% Triton X-100 and 5% donkey serum in TBS). Sections were then incubated with primary antibodies in blocking solution for 16–24 h at 4 °C. Primary antibodies used in this study include anti-PAX6 (BioLegend, 90130, 1:200 dilution), BLBP (Abcam, ab32423, 1:200 dilution), ZEB2 (Origene, TA802113, 1:150 dilution), ZO1 (BD Biosciences, 610966, 1:300 dilution), GFP (R&D systems, AF4240, 1:200 dilution), HSP60 (ptglab, 15282-1-AP, 1:200 dilution), VIM(V9) (Thermo Fisher, MA5-11883, 1:250 dilution), KI67 (Thermo Fisher, 14-5698-82, 1:100 dilution), p53 (7F5), (Cell Signalling, 2527S, 1:500 dilution), OCT3/4 (SCBT; sc-5279, 1:100 dilution), SOX2(E4) (SCBT, sc-365823, 1:50 dilution) and RSL24D1 (Proteintech, 25190-1-AP, 1:100 dilution). After primary antibody, sections were washed with TBS three times with 15 min each before incubating with secondary antibodies in blocking solution for 2 h at RT. Sections were then washed three times with TBS for 15 min each. Sections were then incubated with Hoechst 33342 at 2 µg ml^−1^ for a 15-min incubation at RT. Slides were mounted with ProLong Gold Antifade Mountant (Thermo Fisher, P36930). Stained organoid cryosections were imaged using a ZEISS LSM 800 confocal microscope at 0.7–0.8-µm intervals using a ×40 magnification objective. Images were processed using Fiji. For whole-mount imaging, organoids were washed and fixed the same as cryosection. Organoids were permeabilized in blocking solution for 16–24 h at 4 °C, and then incubated with primary antibody in blocking solution for 16–24 h at 4 °C. Organoids were then washed in PBS/0.04% BSA three times for 15 min. Organoids were then incubated with secondary antibodies in blocking solution for 2 h at RT. Optionally, Hoechst 33342 was added into secondary antibody incubation at 2 µg ml^−1^. Organoids were washed three times with PBS/0.04% BSA for 15 min each and then stored in PBS at 4 °C before imaging. Stained organoid cryosections were imaged using a ZEISS LSM 780 confocal microscope at 8–12-µm intervals using a ×20 magnification objective. The images were further processed using Fiji.

### TUNEL assay

Cryosectioning for the TUNEL assay was the same as previously described for IF. Sections were warmed up, washed with PBS twice for 5 min each and were then permeabilized in PBS/0.2% Triton/0.5%BSA for 30 min. After permeabilization, sections were washed with PBS twice for 5 min each. The TUNEL assay reaction was carried out with TUNEL Assay kit, fluorescence 594 nm (Cell Signalling, 48513) according to the manufacturer’s directions.

### OP-Puro-click

One day before labelling, the medium of iPS cells, EBs and organoids was changed to ensure adequate nutrient levels. On the day of labelling, the culture was changed with medium containing 20 µM OPP (Clickchemistrytools, 1407). Samples were incubated at 37 °C for 30 min and were then processed according to the downstream assay. For the whole-mount sample, EBs were fixed as described in the IF section. After fixation, EBs were permeabilized in DPBS/0.25% Triton X-100 for 20 min and were washed once with DPBS/3% BSA before the click reaction. For cryosectioned samples, sections were warmed up, washed with DPBS three times for 5 min each, permeabilized in DPBS/0.25% Triton X-100 for 20 min and washed once with DPBS/3% BSA before the click reaction. The click reaction was carried out with a Click-&-Go Cell Reaction Buffer kit (Clickchemistrytools, 1263) with AZDye 568 Azide (Clickchemistrytools, 1291) according to the manufacturer’s directions.

### Tandem mass tag mass spectrometry

For protein lysate, iPS cells, EBs and organoids were scraped or collected directly by pelleting at 500*g* for 5 min. Samples were washed once with DPBS, and the pellet were snap-frozen at −80 °C until lysis. Samples were lysed in RIPA buffer (Thermo Fisher, 89900) with 0.5 µl ml^−1^ Benzonase (Sigma, E1014) for 15 min on ice. Lysate was centrifuged at 15,000*g* for 15 min at 4 °C. The supernatant was collected and submitted to University of Texas Southwestern (UTSW) Proteomics Core for TMT-18plex. Data were analysed using Proteome Discoverer 3.0 and was searched using the human protein database from UniProt. The mass spectrometry proteomics data have been deposited on the ProteomeXchange Consortium via the PRIDE partner repository^62^ with the dataset identifier PXD063298.

### Dissociation of brain organoids and scRNA-seq

Organoids were dissociated as previously described^63^. In brief, iPS cells and EBs were dissociated in TrypLE (Thermo Fisher, 12605010). Organoids on day 10, 15 and 30 were dissociated in neural dissociation kit-P (Miltenyi Biotec, 130-092-628). The scRNA-seq libraries were generated using the Chromium Single Cell 3′ Library & Gel Bead kit v3 or v3.1 (10x Genomics, PN-1000075, PN-1000121). For each sample, an estimated 10,000 cells were loaded. Libraries were sequenced on an N2K2.

### scRNA-seq data processing

Reads from scRNA-seq were aligned to the GRCh38 human reference genome and the cell-by-gene count matrices were produced using the Cell Ranger pipeline (10x Genomics). Data were analysed using the Seurat R package v.3.1.5 using R v.4.2. Data from ten individual runs were merged. Cells were filtered on the basis of unique molecular identifier (UMI) counts (>500), the number of detected genes (>500), the log_10_ genes per UMI (>0.8) and the fraction of mitochondrial genes (<0.2). UMI counts were normalized for each cell by NormalizeData () with a scale factor of 10,000. We selected the union of the 100 most variable genes for each time point separately (local) as well as across the full dataset (global). The local and global sets were then combined with a previously reported organizer set^36^. Cell-cycle-related genes were excluded from the set based on AnnotationHub. The data were then scaled and the cell cycle scores and mitochondrial scores were regressed out by ScaleData (). Principal-component analysis (PCA) was performed using the Seurat function RunPCA (). The first ten principal components were used to integrate the different time points and genotypes in the dataset using Harmony with max iteration as 50. We performed UMAP using RunUMAP () with the reduction method as ‘harmony’ and otherwise default settings. Cells were clustered in PCA space using FindNeighbors (), followed by FindClusters with resolution of 0.8. Differentially regulated genes in each cluster were identified using FindAllMarkers () with a log(fold change) threshold of 0.25. The top significant differentially regulated genes were selected for heatmap visualization. The features used for integration can be found in Supplementary Table 8. The ribosome biogenesis gene set used in the analysis is listed in Supplementary Table 9.

### Ribo-seq sample preparation and data analysis

Single wild-type and *AIRIM*^*V190G*^ brain organoids were collected in a 0.5-ml tube and subjected to cell lysis by freeze and thaw in the presence of Ribo-ITP lysis buffer. The resulting homogenized cells were then treated with RNase I at 37 °C for 15 min, and the reaction was stopped by adding 2% SDS. The remaining steps of Ribo-ITP experiments were performed as previously described^38^. Ribosome profiling sequencing libraries were prepared using the D-Plex Small RNA-seq kit (C05030001, Diagenode) with slight modifications. The dephosphorylation reaction was supplemented with 0.5 μl T4 PNK (NEB) and the reaction was incubated for 25 min. Subsequently, complementary DNA (cDNA) was amplified for 12 PCR cycles. We used AMPure XP bead cleanup (1.8×), followed by size selection using 3% agarose, dye-free gel cassettes with internal standards (BDQ3010, Sage Science) on the BluePippin platform. Sequencing was performed using an Illumina Novaseq 6000.

Ribosome profiling data were processed using RiboFlow with the following modifications^64^. UMIs corresponding to the first 12 nucleotides from the 5′ end of each read was extracted using UMI-tools^65^. The four nucleotides following the UMI were discarded as they are incorporated during the template-switching reverse transcription step. Transcriptome-mapping reads with mapping quality greater than two were retained using the default settings in RiboFlow. PCR duplicates were removed using UMIs. Finally, ribo files were generated using RiboFlow and further analyses, including metagene plots and quality control were generated using RiboR^64^.

### Cytoplasmic RNA-seq sample preparation and data analysis

Organoids were produced with bulk-embedding as described above (Generating cerebral organoids). On day 10, 300× organoids were collected and were dissociated from Matrigel by centrifuging at 500*g* for 5 min. To extract cytoplasmic RNA, the organoid pellet was lysed in lysis buffer (20 mM Tris-HCl, pH 7.5, 5 mM MgCl_2_, 150 mM NaCl, 1 mM dithiothreitol (DTT) and 0.1% NP-40). The lysate was collected into a 1.5-ml tube and incubated on ice for 15 min. The lysate was then centrifuged at 21,130*g* at 4 °C for 10 min to remove nuclei, mitochondria and debris. The supernatant was collected and cytoplasmic RNA was extracted by Trizol-LS (Thermo Fisher, 10296028) following the manufacturer’s protocol. Strand-specific whole-transcriptome (ribo-minus) sequencing was performed at the McDermott Center Next-Generation Sequencing (NGS) Core at UTSW using a TruSeq Stranded mRNA Sample Preparation kit (Illumina). The reads were then used as the reference of single-organoid ribo-seq under same conditions. The 5′ adaptor sequence ‘AGATCGGAAGAGCACACGTCTGAACTCCAGTCA’ was clipped from the first read and processed using RiboFlow to quantify read count reads per transcript.

### Differential translation efficiency analysis

Using RiboR, we extracted read counts corresponding to coding regions from the ‘ribo’ files across all experiments. All analyses utilized ribosome footprints ranging 26–33 nucleotides in length. We performed a joint analysis and normalization of ribosome occupancy and RNA-seq data employing the TMM normalization method^66^. We then calculated transcript-specific dispersion estimates and assessed differential translation efficiency using the edgeR tool^67^. Specifically, to identify genes with differential translation efficiency, we evaluated changes in ribosome profiling while adjusting for differences in RNA expression using a generalized linear model, in which we considered RNA expression and ribosome occupancy as two experimental manipulations of the cell’s RNA pool^68^. We employed the Benjamini–Hochberg procedure to compute adjusted *P* values. Motifs enriched in the 5′ UTRs of *AIRIM*^*V190G*^-sensitive transcripts were analysed. *P* values were calculated based on the hypergeometric distribution (two-sided, multiple-comparison, corrected test)^69^.

### Gene-set enrichment analysis

For the gene-set enrichment analysis shown in Fig. 7c, we performed the Correlation Adjusted MEan RAnk (CAMERA) method^70^, with results visualized using Cytoscape^71^. We used pre-ranked CAMERA analysis using all genes ordered by their TE values. Genes were ranked by their log_2_(fold change) values, where negative values indicate decreased translation efficiency in mutant compared with control organoids. We performed enrichment against the Gene Ontology (GO) cellular component (CC) gene sets from MSigDB (v.2023.1), filtering to include only gene sets containing between 10 and 500 genes, to ensure statistical robustness, while avoiding overly broad categories. The analysis revealed that the significantly enriched GO CC terms (false discovery rate (FDR) < 0.01) were predominantly in the ‘down’ direction. The network visualization shows 14 representative gene sets selected from 25 total significant gene sets (FDR < 0.01). Four terms (GOCC_POLYSOMAL_RIBOSOME, GOCC_TRANSLATION_PREINITIATION_COMPLEX, GOCC_POLYSOME and GOCC_CHAPERONE_COMPLEX) were omitted to reduce redundancy and improve visual clarity. Another seven terms that overrepresented among genes with increased TE (direction, up) in mutant organoids (GOCC_CENTRIOLE, GOCC_SYNAPTIC_MEMBRANE, GOCC_POSTSYNAPTIC_DENSITY_MEMBRANE, GOCC_PRESYNAPTIC_MEMBRANE, GOCC_CILIARY_BASAL_BODY, GOCC_POSTSYNAPTIC_MEMBRANE and GOCC_RECEPTOR_COMPLEX) were not presented as they did not cluster into a meaningful network.

The complete CAMERA analysis output, including FDR values for each gene set and the genes within each gene set, is provided in Supplementary Table 10. The FDR of 14 presented gene sets are listed in Supplementary Table 11. The edges in the network represent similarity between pathway terms, calculated based on shared genes between nodes by cytoscape. Edge weight represents the strength of similarity, with a cutoff set at 0.5 (similarity scores ranging 0.375–1.0, with denser connections indicating stronger similarity). We also performed parallel analyses using GO Biological Process (BP) and general GO gene sets, which validated our findings. To reduce redundancy, we presented GO CC results as representative. The CAMERA results (GO BP and GO) are now available as Supplementary Tables 12 and 13, respectively.

### Seahorse analysis

Human cerebral organoids (hCOs) were dissected from Matrigel, the day before the experiment, each well was plated with approximately 15,000 cells (5× organoids). On the day of the assay, the cell culture medium was removed and replaced with Seahorse assay medium supplemented with glutamine. The cells were allowed to acclimate to the assay medium for 2 h in the incubator. Once acclimated, the cells were assayed for basal OCR, ATP-dependent OCR and maximal OCR using a standard mitochondrial stress test protocol. For the assay, 2 µM oligomycin and 1 µM FCCP were used for the injections. After the assay was completed, cells were collected and total protein levels were measured using the BCA protein assay to ensure that the observed changes in respiration were not due to variations in cell number. All experiments were conducted on a Seahorse XFp machine.

### Transmission electron microscopy

On day 15, 20× organoids were collected for TEM images. Organoids were rinsed in pre-chilled PBS, and then fixed with 2.5% (v/v) glutaraldehyde in 0.1 M sodium cacodylate buffer for 1 h at RT. Next, the samples were rinsed three times with 0.1 M sodium cacodylate buffer. Then, the organoids were post-fixed in 1% osmium tetroxide for 1 h at RT. After fixation, the samples were rinsed with PBS three times and stained with 2% aqueous uranyl acetate for 1 h. Specimens were rinsed with distilled water five times and dehydrated with a 50%, 70% and 90% ethanol concentration series infiltrated with Embed-812 resin. Beem capsules were overfilled with resin and polymerized in a 60 °C oven overnight. The section thickness was adjusted to 100 nm. Beem capsule blocks were sectioned with a diamond knife (Diatome) on a Leica Ultracut UCT ultramicrotome (Leica Microsystems) and collected onto copper grids. The sections were post-stained with 2% uranyl acetate in water and lead citrate. Images were acquired on a JEOL 1400+ transmission electron microscope (FEI) equipped with a LaB6 source.

### Nuclear and cytoplasmic partitioning

For iPS cells, cells were collected grown to 80% confluency in a 100-mm dish. Organoids were produced with bulk-embedding as described for generating cerebral organoids. On day 15, 300× organoids were collected and were dissociated from Matrigel by centrifuging at 500*g* for 5 min. To extract the cytoplasmic fraction, cells or organoid pellets were lysed in lysis buffer (20 mM Tris-HCl, pH 7.5, 5 mM MgCl_2_, 150 mM NaCl, 1 mM DTT and 0.1% NP-40). The lysate was collected in a 1.5-ml tube and kept on ice for 15 min. The lysate was then centrifuged at 21,130*g* at 4 °C for 10 min to remove nuclei, mitochondria and debris. The supernatant was collected for the polysome fractionation.

### Polysome profiling and fractionation

We prepared a sucrose gradient solution using ultra-pure DNase/RNase-free H_2_O (Invitrogen) or DEPC-treated H_2_O. For the 0% solution, we mixed 1 ml of 1 M Tris (pH 7.5), 250 µl of 1 M MgCl_2_ and 1 ml of 5 M NaCl, and then added RNase-free H_2_O to a final volume of 50 ml. For the 60% solution, we dissolved 30 g of DNase/RNase-free sucrose in 1 ml of 1 M Tris (pH 7.5), 250 µl of 1 M MgCl_2_ and 1.5 ml of 5 M NaCl, and then supplemented with RNase-free H_2_O to a final volume of 50 ml. We created a 10%–50% sucrose gradient from the 0% and 60% solutions. We quantified the RNA concentration of the lysates using a Nanodrop and loaded 200 µg of RNA onto the 10%–50% sucrose gradient. The sample was centrifuged in a TH-641 rotor (Thermo Scientific) at 34,000 rpm for 2 h at 4 °C, fractionated and the gradients were collected using a BioLogic LP Chromatography System (Bio-Rad). We precipitated the proteins in the fractions with methanol, then resuspended them in 4× Laemmli buffer (Bio-Rad, 1610747) containing 2-mercaptoethanol (Bio-Rad, 1610710) and denatured them at 95 °C for 10 min.

### Western blotting

Denatured samples of equal amounts were loaded onto stain-free 4–15% Mini-PROTEAN TGX Precast Protein Gels (Bio-Rad, 4561086) for separation at 100 V for 1.5 h. Total proteins were then transferred to a Hybond 0.45 µm PVDF blotting membrane (Cytiva, 10600029) at 0.3 A for 1 h on ice. Blots were blocked with 8% non-fat milk in TBS buffer at RT and incubated with primary antibodies overnight at 4 °C. The membrane was rinsed with TBST for three 10-min washes and then incubated with peroxidase-conjugated secondary antibodies at RT for 1 h. The membrane was rinsed with TBST for three 10-min washes and imaged using the Bio-Rad ChemiDoc Touch Imaging System.

The primary antibodies used were rabbit anti-RSL24D1 (Proteintech, 25190-1-AP, 1:500 dilution); rabbit anti-RPL28 (Abcam, ab138125, 1:1,000 dilution); rabbit anti-TSC1 (D43E2) (CST, 6935,1:1,000 dilution); and mouse anti-GAPDH (6C5) (Millipore, MAB374, 1:1,000 dilution).

### Site-directed mutagenesis, transfection and immunoprecipitation

Expression vectors were generated by cloning cDNA (Biosettia, cDNA-has-03) (AIRIM Refseq NM_017850.3) into the pOZ-N–Flag–HA retroviral vector using standard protocols. Mutants were generated using a Q5 site-directed mutagenesis kit (NEB, E0554S) and confirmed by Sanger sequencing. Transient plasmid transfection was carried out with Lipofectamin 2000 (Invitrogen, 11668019), according to the manufacturer’s instructions. After 48 h transfection of plasmids, cells were collected and subjected to immunoprecipitation. Immunostaining with anti-Flag M2-agarose beads was performed as previously described^16^ with slight modifications. For whole-cell co-immunoprecipitation, cells were lysed in immunoprecipitation buffer (100 mM NaCl, 50 mM Tris-HCl, pH 7.4, 0.2 % NP-40, 10 % glycerol, 1 mM MgCl_2_ and 1 mM DTT) supplemented with complete EDTA-free protease inhibitor cocktail (Roche) and 50 U ml^−1^ Benzonase (Sigma, 70746-3) and incubated at 4 °C for 1 h under end-over-end rotation. After Benzonase digestion, lysates were cleared by centrifugation. Flag IP was performed using anti-Flag M2-agarose beads (Sigma, A2220) for 3 h and at 4 °C followed by Flag peptide (Sigma, F4799) for elution overnight at 4 °C. Elutions were subjected to western blotting. The primary antibodies used were rabbit anti-HA (CST, 3274); rabbit anti-POLD3 (Abcam, 182564); rabbit anti-AFG2A (Abcam, ab189519); rabbit anti-AFG2B (Novus, NBP1-92430); and rabbit anti-CINP (Abcam, 180955).

### AlphaFold 3D structure prediction

To compare the structures of wild-type and mutant AIRIM variants, corresponding models were predicted in the context of the 55LCC ‘Lid’ unit^16^. Protein sequences spanning the folded N-terminal domains of AFG2A (residues 45–334) and AFG2B (residues 12–192), full-length CINP (residues 1–212) and full-length AIRIM variants (residues 1–203) were used as inputs in AlphaFold 3 (ref. ^72^), assuming a 4:2:2:2 stoichiometry^16^. Structural predictions were run using the default settings on the AlphaFold 3 webserver, asking for five models as outputs. All structural predictions were manually inspected and models with the highest-ranking score (usually between 0.74–0.76) were superimposed in UCSF Chimera X (v.1.6.1)^73,74^, using one of the AIRIM protomer in the predicted wild-type structure as a reference.

### Structure visualization

Structural representations depicted in Extended Data Fig. 1a,b were created in UCSF Chimera X (v.1.6.1)^73,74^.

### Statistics and reproducibility

No statistical methods were used to predetermine sample sizes. Sample size was determined based on previous studies in the field^29,37^. Data collection and analysis were not performed blind to the conditions of the experiments. Data distribution was assumed to be normal but this was not formally tested. No randomization in the organization of the experimental conditions or stimulus presentation was used. No data points were excluded from the analyses for any reason.

#### Organoid size analysis

For AIRIM across time points. For day 5: *n* = 73 for total control EBs, *n* = 62 for total mutant EBs, from three experimental batches. For day 10: *n* = 77 for total control organoids, *n* = 84 for total mutant organoids, from four experimental batches. For day 15: *n* = 46 for total control organoids, *n* = 51 for total mutant organoids, from four experimental batches. For day 30: *n* = 12 for total control organoids, *n* = 12 for total mutant organoids, from two experimental batches. *P* values were calculated using an unpaired *t*-test with Welch’s correction. For *TSC1*: *n* = 11 for total control *TSC1*+/+ organoids, *n* = 12 for total *V190G TSC1*+/+ organoids, *n* = 37 for total control *TSC1* +/− organoids, *n* = 35 for total *V190G TSC1*+/− organoids, from two experimental batches. *P* values were calculated using Dunnett’s T3 multiple comparisons test.

#### Proteomic analysis

For each batch, 300 EBs, 300 day 10 organoids and 100 day 15 organoids were used for control and V190G mutant. Samples from three individual batches were analysed. To detect the abundance of ribosomal proteins across conditions, a repeated measures one-way analysis of variance and Tukey’s multiple comparison were performed.

#### scRNA-seq analysis

Fifty day 5 EBs, 3–6 day 10 NE, 3–6 day 15 organoids and 3 day 30 organoids of each genotype were pooled for each dissociation. For each condition, approximately 10,000 cells were sequenced.

#### Single-organoid ribo-seq analysis

Two control and three mutant organoids on day 10 were sequenced.

#### Bulk RNA-seq analysis

A total of 300 organoids were pooled and sequenced per genotype, per replicate, with a total of two biological replicates.

#### Immunofluorescence, TUNEL and OP-Puro imaging

Twelve organoids of each condition were used for the assays. *P* values were calculated from Welch’s *t*-test (two conditions) or Kruskal–Wallis test (multiple comparison).

#### Mitochondrial aggregation quantification

Images were processed via ImageJ (Fiji): subtract background (radius = 50), Gaussian blur (radius = 3.00), threshold-dark background and analyse particles (size 5–100 μm^2^).

The number of particles (aggregated mitochondria signal) was then normalized by area (mm^2^) of the imaged NE bud. The centre of NE bud was manually removed to avoid signal artifacts caused by debris.

### Reporting summary

Further information on research design is available in the Nature Portfolio Reporting Summary linked to this article.

## Online content

Any methods, additional references, Nature Portfolio reporting summaries, source data, extended data, supplementary information, acknowledgements, peer review information; details of author contributions and competing interests; and statements of data and code availability are available at 10.1038/s41556-025-01708-8.

## Supplementary information

Reporting Summary

Supplementary Tables Supplementary Tables 1–13.

## Source data

Source Data Fig. 4 and Extended Data Figs. 1, 5 and 8 Unprocessed western blots.

Source Data Figs. 3, 4, 6–8 and Extended Data Figs. 2–8 Numerical source data for all relevant figures and extended data figures.

## Data Availability

Sequencing data that support the findings of this study have been deposited in the Gene Expression Omnibus under accession code GSE247456. All analysis was conducted on the human genome version hg38. Proteomics datasets have been deposited and are available at the ProteomeXchange Consortium under accession code PXD063298. Other forms of source data are provided in this study. Reagents generated or any other information supporting the findings of this study are available from the corresponding authors on reasonable request. Source data are provided with this paper.
