# Supplementary material for: A programmed decline in ribosome levels governs human early neurodevelopment
Source: Nat Cell Biol. 2025 Aug 4;27(8):1240–55. doi: 10.1038/s41556-025-01708-8 (PMC12339376; doi:10.1038/s41556-025-01708-8)
Supplement: Supplementary file 1 — Reporting Summary [file 41556_2025_1708_MOESM1_ESM.pdf]

nature portfolio

Corresponding author(s): Professor Michael Buszczak , Professor Leqian Yu,
Professor Jun Wu,
Reza Maroofian

Last updated by author(s): May 29, 2025

Reporting Summary

Nature Portfolio wishes to improve the reproducibility of the work that we publish. This form provides structure for consistency and transparency in reporting. For further information on Nature Portfolio policies, see our Editorial Policies and the Editorial Policy Checklist.

Statistics

For all statistical analyses, confirm that the following items are present in the figure legend, table legend, main text, or Methods section.

n/a	Confirmed	
☐	☒	The exact sample size (*n*) for each experimental group/condition, given as a discrete number and unit of measurement
☐	☒	A statement on whether measurements were taken from distinct samples or whether the same sample was measured repeatedly
☐	☒	The statistical test(s) used AND whether they are one- or two-sided<br>*Only common tests should be described solely by name; describe more complex techniques in the Methods section.*
☐	☒	A description of all covariates tested
☐	☒	A description of any assumptions or corrections, such as tests of normality and adjustment for multiple comparisons
☐	☒	A full description of the statistical parameters including central tendency (e.g. means) or other basic estimates (e.g. regression coefficient) AND variation (e.g. standard deviation) or associated estimates of uncertainty (e.g. confidence intervals)
☐	☒	For null hypothesis testing, the test statistic (e.g. *F*, *t*, *r*) with confidence intervals, effect sizes, degrees of freedom and *P* value noted<br>*Give P values as exact values whenever suitable.*
☒	☐	For Bayesian analysis, information on the choice of priors and Markov chain Monte Carlo settings
☒	☐	For hierarchical and complex designs, identification of the appropriate level for tests and full reporting of outcomes
☒	☐	Estimates of effect sizes (e.g. Cohen's *d*, Pearson's *r*), indicating how they were calculated

Our web collection on statistics for biologists contains articles on many of the points above.

Software and code

Policy information about availability of computer code

Data collection	Proteome Discoverer 3.0 for proteomic data acquisition, ZEN 2.1 (blue edition) for microscopic images acquisition.
Data analysis	Cell Ranger 7.0.0 for scRNA-seq, R v.4.2 for multiple analyses including: Seurat Rpackage v.3.1.5 for scRNA-seq, RiboR for ribo-seq, and edgeR v3 for RNA-seq. FIJI forimage analysis. GraphPad Prism 8.2.0 for statistical analysis.

For manuscripts utilizing custom algorithms or software that are central to the research but not yet described in published literature, software must be made available to editors and reviewers. We strongly encourage code deposition in a community repository (e.g. GitHub). See the Nature Portfolio guidelines for submitting code & software for further information.

Data

Policy information about availability of data

All manuscripts must include a data availability statement. This statement should provide the following information, where applicable:

- Accession codes, unique identifiers, or web links for publicly available datasets
- A description of any restrictions on data availability
- For clinical datasets or third party data, please ensure that the statement adheres to our policy

Sequencing data that support the findings of this study have been deposited in the Gene Expression Omnibus (GEO) under accession code GSE247456. All analysis was done on human genome version hg38. Proteomics datasets have been deposited and are available at the ProteomeXchange Consortium under accession code

PXD063298. Other forms of source data are provided in this study. Reagents generated or any other information supporting the findings of this study are available from the corresponding author on reasonable request.

Research involving human participants, their data, or biological material

Policy information about studies with human participants or human data. See also policy information about sex, gender (identity/presentation), and sexual orientation and race, ethnicity and racism.

Reporting on sex and gender	We describe 11 families with allelic variants in AIRIM. Sex was assigned and reported in the appropriate figures and tables. This is not a population study, therefore sex- and gender based analyses were not performed.
Reporting on race, ethnicity, or other socially relevant groupings	Ancestry of the patients was reported by family members.
Population characteristics	No data on population characteristics was collected or used in this study.
Recruitment	N/A
Ethics oversight	Ethical oversight for all human data described in this study was provided by Phoenix Children's Hospital (PCH IRB #15-080), University College London Queen Square Institute of Neurology (22/NE/0080, project ID 310045), Heidelberg University (S-186/2012), King Faisal Hospital Specialist & Research Centre (20DG1533: RAC#2121053 and 23DG0161: RAC# 2210029), National and Kapodistrian University of Athens (16434/25-07-22), and King Abdullah International Medical Research Center (IRB/1470/24, project number NRC23R/177/02).

Note that full information on the approval of the study protocol must also be provided in the manuscript.

Field-specific reporting

Please select the one below that is the best fit for your research. If you are not sure, read the appropriate sections before making your selection.

☒ Life sciences ☐ Behavioural & social sciences ☐ Ecological, evolutionary & environmental sciences

For a reference copy of the document with all sections, see nature.com/documents/nr-reporting-summary-flat.pdf

Life sciences study design

All studies must disclose on these points even when the disclosure is negative.

Sample size	Proteomic analysis: For each batch, 300 EBs, 300 day 10 organoids and 100 day 15 organoids were used for control and V190G mutant. Samples from three individual batches were analyzed, as a higher degree of accuracy was desired than typical in the field for detecting quantitative changes at individual proteins across organoid development.<br>scRNAseq: 50 day 5 embryoid bodies, 3-6 day 10 neuroepithelia, 3-6 day 15 organoids and 3 day 30 organoids of each genotype were pooled for each dissociation. For each condition, approximately 10,000 cells were sequenced, similar to other studies in the field, where large effects can be easiliy identified at this sample size.<br>Single organoid ribo-seq: Two control and three mutant day 10 organoids were used, where large effects can be easiliy identified at this sample size.<br>Bulk RNA-seq: 300 organoids were pooled and sequenced per genotype, per replicate, with a total of 2 biological replicates, where large effects can be easiliy identified at this sample size.<br>Organoid size analysis: a minimum of n=2 individual batches were performed, with mesaurements on multiple individual organoids per experiments.<br>Immunofluorescence/TUNEL/OP-Puro imaging/Mitochondrial aggregation: a miniimum of n=3 individual oragnoid staining was performed for quantitative analysis.<br>No statistical methods were used to predetermine sample sizes. Sample size was determined based on previous studies in the field(Lancaster, M. A. et al. Cerebral organoids model human brain development and microcephaly. Nature 501, 10.1038/nature12517 (2013), Benito-Kwiecinski, Silvia et al. Cell, Volume 184, Issue 8, 2084 - 2102.e19 An early cell shape transition drives evolutionary expansion of the human forebrain).
Data exclusions	No data were excluded from imaging-based analysis, scRNA-seq, and ribo-seq.
Replication	Western blots, sea-horse, electron microscopy and teratoma assays were runat least twice, independently, with similar results. Organoid size quantification, pH3 counting, TUNEL quantification, OP-puro quantification and proteomic analysis were performed on at least two independent biological replicates with similar results.
Randomization	Samples were assigned to groups based on genotype and treatment conditions.
Blinding	No blinding was performed. All samples were processed and analyzed equally.

Reporting for specific materials, systems and methods

We require information from authors about some types of materials, experimental systems and methods used in many studies. Here, indicate whether each material, system or method listed is relevant to your study. If you are not sure if a list item applies to your research, read the appropriate section before selecting a response.

Materials & experimental systems

n/a	Involved in the study
☐	☒ Antibodies
☐	☒ Eukaryotic cell lines
☒	☐ Palaeontology and archaeology
☐	☒ Animals and other organisms
☒	☐ Clinical data
☒	☐ Dual use research of concern
☒	☐ Plants

Methods

n/a	Involved in the study
☒	☐ ChIP-seq
☒	☐ Flow cytometry
☐	☒ MRI-based neuroimaging

Antibodies

Antibodies used

PAX6 (BioLegend, 90130, 1:100)
BLBP (Abcam, ab32423, 1:100)
ZEB2(OTI1E12) (OriGene, TA802113, 1:100)
ZO1(1/ZO-1) (BD Biosciences, 610966, 1:100)
GFP (R&D Systems, AF4240, 1:100)
HSP60 (Proteintech, 15282-1-AP, 1:100)
Vimentin(V9) (Thermo Fisher, MA5-11883, 1:100)
KI67(SolA15) (Thermo Fisher, 14-5698-82, 1:100)
p53(7F5) (Cell Signaling, 2527S, 1:100)
OCT3/4(C-10) (Santa Cruz, sc-5279, 1:100)
SOX2(E4) (Santa Cruz, sc-365823, 1:100)
RSL24D1 (Proteintech, 25190-1-AP, 1:100 for IF)
RSL24D1 (Proteintech, 25190-1-AP, 1:500 for WB)
RPL28 (Abcam, ab138125, 1:1000 for WB)
TSC1(D43E2) (Cell Signaling, 6935, 1:1000 for WB)
GAPDH(6C5) (Millipore, MAB374, , 1:1000 for WB)
Secondary antibodies:
Donkey-anti-Mouse Alexa 488, 568 conjugated secondary (Thermofisher A-21202, A10037, 1:250);
Donkey-anti-Rabbit Alexa 488 conjugated secondary (Thermofisher A-21206, A10042, 1:250);
Donkey-anti-Rat Alexa 488, 568 conjugated secondary (Thermofisher A-21208, 1:250);
Donkey-anti-Goat Alexa 488, 568, 647 conjugated secondary antibodies(Thermofisher, A-11055, A-11057, A-21447, 1:250).
Peroxidase IgG Fraction Monoclonal Mouse Anti-Rabbit IgG, light chain specific, (Jackson ImmunoResearch, 211-032-171, 1:5000).
Peroxidase AffiniPure Donkey Anti-Mouse IgG (H+L) (Jackson ImmunoResearch, 715-035-150, 1:1000).

Validation

PAX6 (BioLegend, 90130, 1:100): Validated by BioLegend and used in 337 scientific literatures.
BLBP (Abcam, ab32423, 1:100): Validated by Abcam and used in 99 scientific literatures.
ZEB2 (OTI1E12) (OriGene, TA802113, 1:100): Validated by OriGene and used in 8 scientific literatures.
ZO1 (1/ZO-1) (BD Biosciences, 610966, 1:100): Validated by BD Biosciences and used in 83 scientific literatures.
GFP (R&D Systems, AF4240, 1:100): Validated by R&D Systems and used in 17 scientific literatures.
HSP60 (Proteintech, 15282-1-AP, 1:100): Validated by Proteintech and used in 149 scientific literatures.
Vimentin (V9) (Thermo Fisher, MA5-11883, 1:100): Validated by Thermo Fisher and used in 246 scientific literatures.
KI67 (SolA15) (Thermo Fisher, 14-5698-82, 1:100): Validated by Thermo Fisher and used in 414 scientific literatures.
p53 (7F5) (Cell Signaling, 2527S, 1:100): Validated by Cell Signaling Technology and used in 666 scientific literatures.
OCT3/4 (C-10) (Santa Cruz, sc-5279, 1:100): Validated by Santa Cruz Biotechnology and used in 2693 scientific literatures.
SOX2 (E4) (Santa Cruz, sc-365823, 1:100): Validated by Santa Cruz Biotechnology and used in 355 scientific literatures.
RSL24D1 (Proteintech, 25190-1-AP, 1:100): Validated by Proteintech and used in 4 scientific literatures.
RPL28 (Abcam, ab138125): Validated by Abcam and used in 4 scientific literatures.
TSC1 (D43E2) (Cell Signaling, 6935): Validated by Cell Signaling Technology and used in 99 scientific literatures.
GAPDH (6C5) (Millipore, MAB374): Validated by Millipore and used in 428 scientific literatures.

Eukaryotic cell lines

Policy information about cell lines and Sex and Gender in Research

Cell line source(s)

SCVI274 was obtained from the Stanford CVI Biobank. Human ES cell line H9 (WA09) was obtained from WiCell. The HEK293T cell line was obtained from ATCC (American Type Culture Collection)

Authentication

SCVI274 cells and its derivatives were validated by karyotyping by WiCell. Engineered cells were validated by sequencing. H9

Authentication	cells were authenticated by short tandem repeat (STR) profiling. HEK293T cells were validated by confirming their morphology and growth characteristics
Mycoplasma contamination	All lines tested negative for mycoplasma contamination
Commonly misidentified lines (See ICLAC register)	None

Animals and other research organisms

Policy information about studies involving animals; ARRIVE guidelines recommended for reporting animal research, and Sex and Gender in Research

Laboratory animals	Female immunodeficiency NOD-SCID mice (~10 weeks old) were used for teratoma assays. Mice were housed in 12-hr light/12-hr dark cycle 22.1–22.3 °C and 33–44% humidity.
Wild animals	No wild animals were used in the study.
Reporting on sex	Female mice were used in teratoma assays
Field-collected samples	No field collected samples were used in the study.
Ethics oversight	UT Southwestern Institutional Animal Care and Use Committee (IACUC): APN-2018-102430

Note that full information on the approval of the study protocol must also be provided in the manuscript.

Plants

Seed stocks	*Report on the source of all seed stocks or other plant material used. If applicable, state the seed stock centre and catalogue number. If plant specimens were collected from the field, describe the collection location, date and sampling procedures.*
Novel plant genotypes	*Describe the methods by which all novel plant genotypes were produced. This includes those generated by transgenic approaches, gene editing, chemical/radiation-based mutagenesis and hybridization. For transgenic lines, describe the transformation method, the number of independent lines analyzed and the generation upon which experiments were performed. For gene-edited lines, describe the editor used, the endogenous sequence targeted for editing, the targeting guide RNA sequence (if applicable) and how the editor was applied.*
Authentication	*Describe any authentication procedures for each seed stock used or novel genotype generated. Describe any experiments used to assess the effect of a mutation and, where applicable, how potential secondary effects (e.g. second site T-DNA insertions, mosiacism, off-target gene editing) were examined.*

Magnetic resonance imaging

Experimental design

Design type	Retrospective study.
Design specifications	Retrospective review of neuroimaging MRI studies initially acquired for clinical purposes.
Behavioral performance measures	N/A

Acquisition

Imaging type(s)	MRI
Field strength	1.5 and 3.0 TESLA
Sequence & imaging parameters	Due to the number of participating centers in the patient cohort, there was significant heterogeneity in terms of scanner manufacturer, sequences acquired, and imaging parameters. Minimum MR imaging sequences for inclusion were axial T1WI and axial T2WI, all with ≤5-mm section thicknesses. Additional sequences including T2 FLAIR, SWI, DWI/DTI, and gradient recalled echo were reviewed in most cases (when available).
Area of acquisition	Brain

Diffusion MRI ☒ Used ☐ Not used

Parameters	*Specify # of directions, b-values, whether single shell or multi-shell, and if cardiac gating was used.*

Preprocessing

Preprocessing software	N/A
Normalization	*If data were normalized/standardized, describe the approach(es): specify linear or non-linear and define image types used for transformation OR indicate that data were not normalized and explain rationale for lack of normalization.*
Normalization template	*Describe the template used for normalization/transformation, specifying subject space or group standardized space (e.g. original Talairach, MNI305, ICBM152) OR indicate that the data were not normalized.*
Noise and artifact removal	*Describe your procedure(s) for artifact and structured noise removal, specifying motion parameters, tissue signals and physiological signals (heart rate, respiration).*
Volume censoring	*Define your software and/or method and criteria for volume censoring, and state the extent of such censoring.*

Statistical modeling & inference

Model type and settings	N/A
Effect(s) tested	N/A

Specify type of analysis: ☒ Whole brain ☐ ROI-based ☐ Both

Statistic type for inference<br>(See Eklund et al. 2016)	N/A
Correction	N/A

Models & analysis

n/a	Involved in the study
☒	☐ Functional and/or effective connectivity
☒	☐ Graph analysis
☒	☐ Multivariate modeling or predictive analysis
