# Supplementary material for: A programmed decline in ribosome levels governs human early neurodevelopment
Source: Nat Cell Biol. 2025 Aug 4;27(8):1240–55. doi: 10.1038/s41556-025-01708-8 (PMC12339376; doi:10.1038/s41556-025-01708-8)

Related to Figure 4F

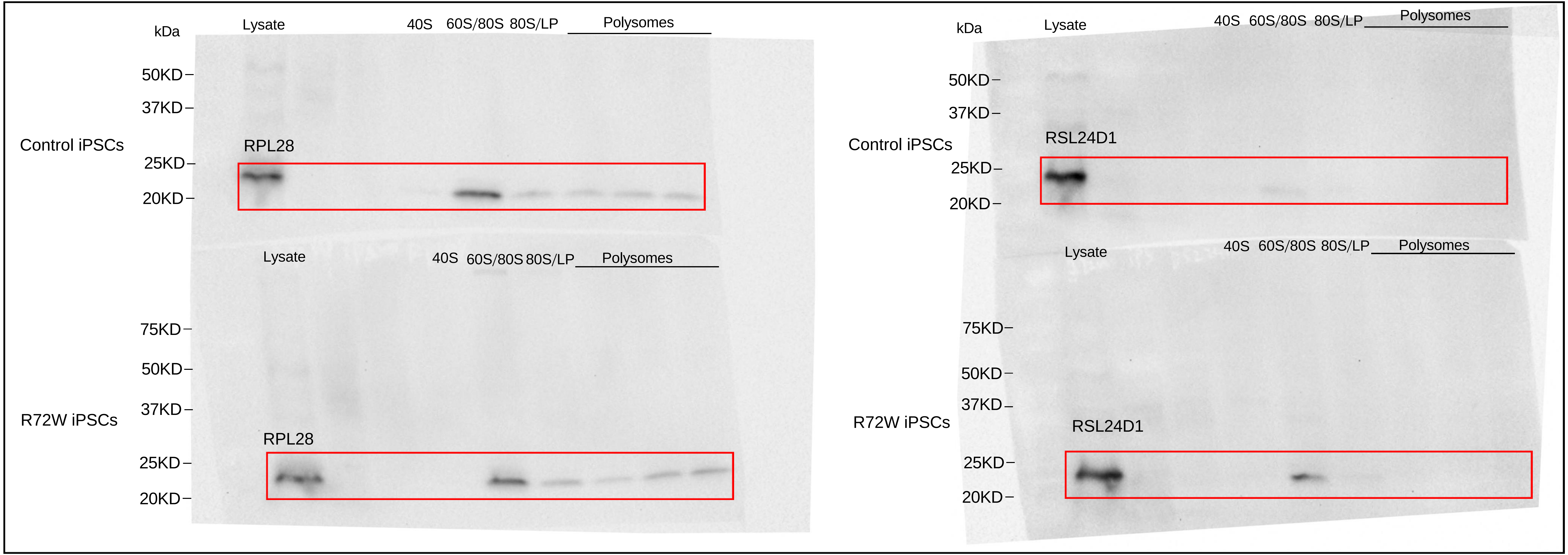

Related to Figure 4G

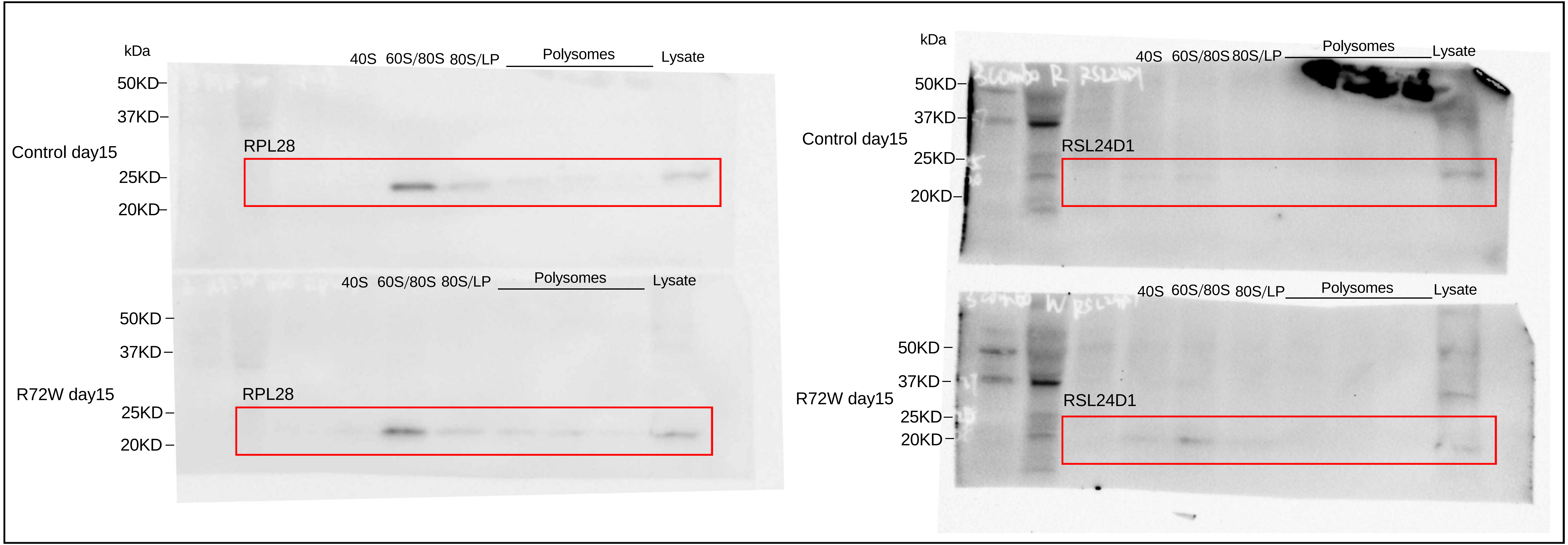

Related to Extended data Figure 1C

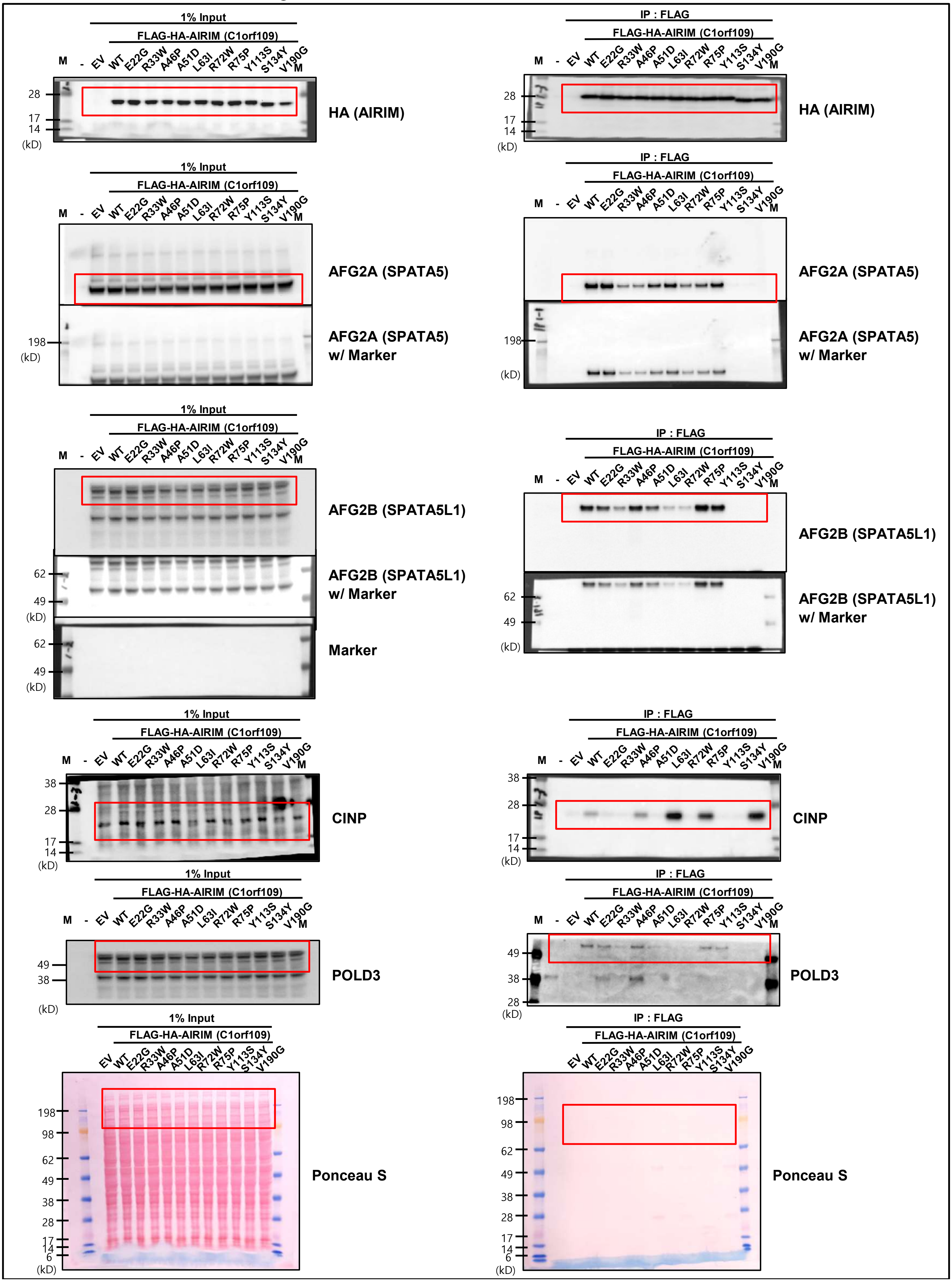

Related to Extended data Figure 5C

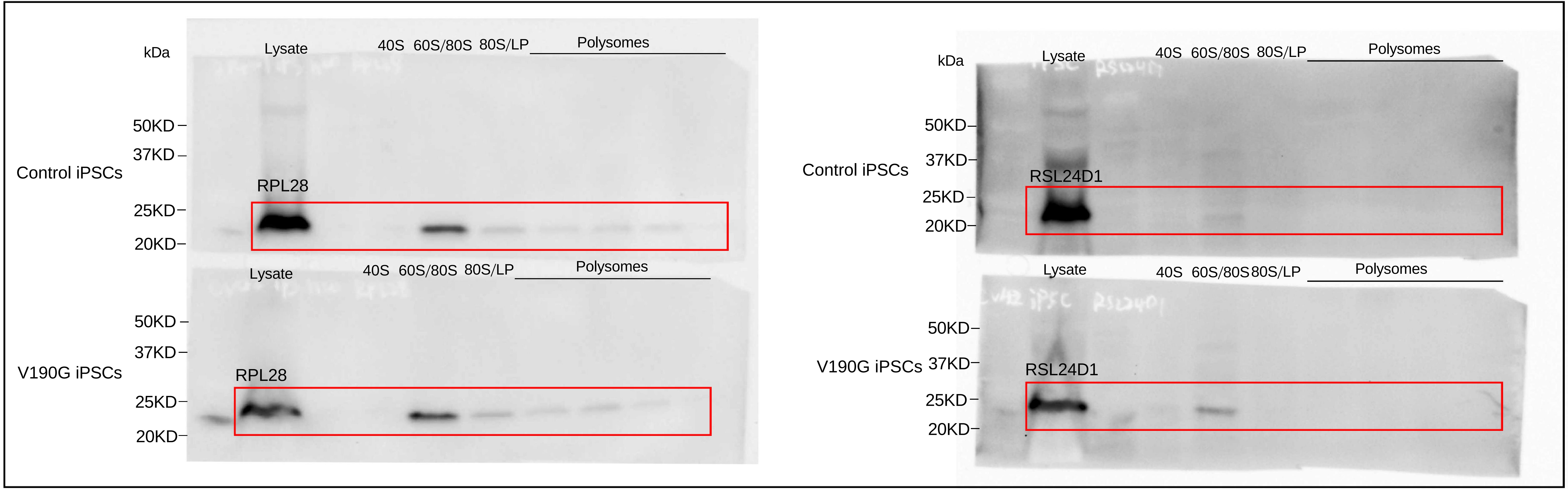

Related to Extended data Figure 5D

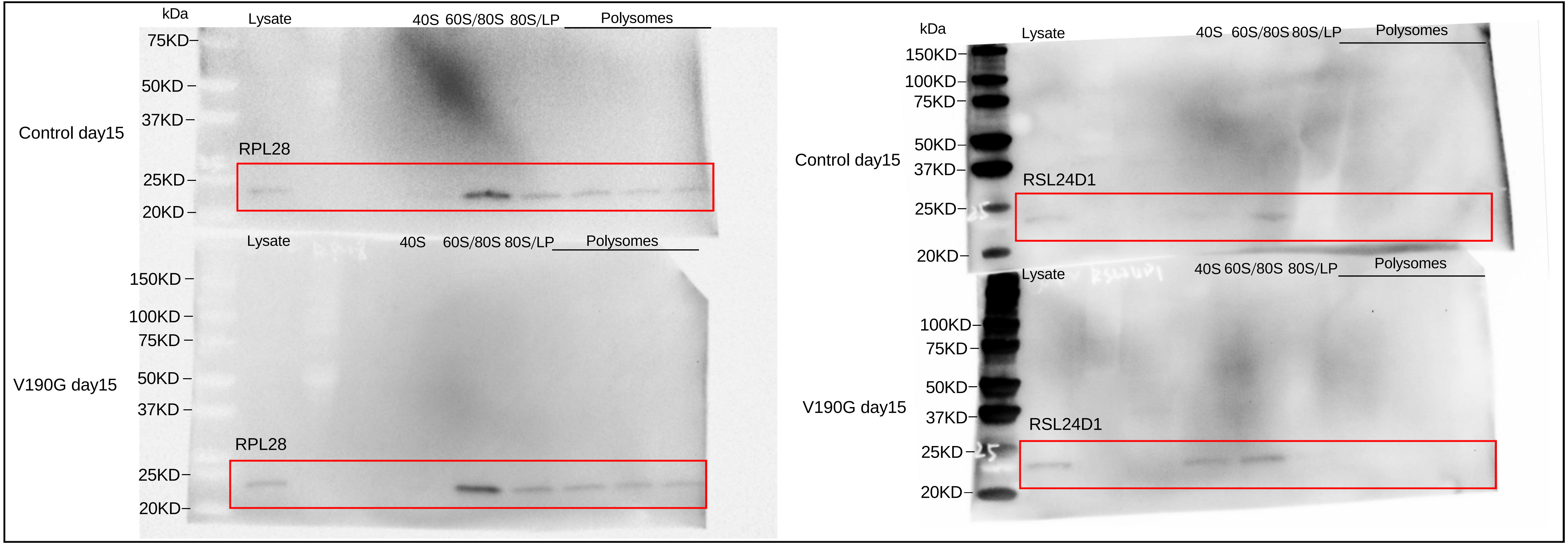

Related to Extended data Figure 8B

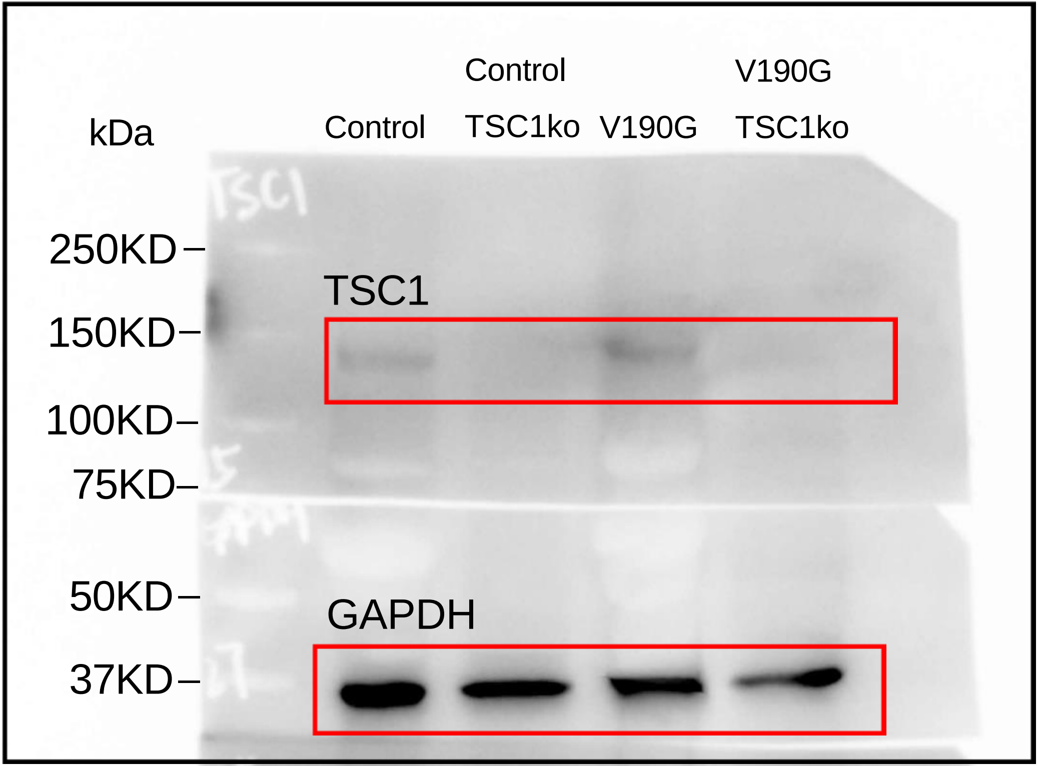

Supplement: Supplementary file 3 — Unprocessed western blots. [file 41556_2025_1708_MOESM3_ESM.pdf]
